# Molecular and cellular mechanisms involved in tissue-specific metabolic modulation by SARS-CoV-2

**DOI:** 10.3389/fmicb.2022.1037467

**Published:** 2022-11-10

**Authors:** Alef Aragão Carneiro dos Santos, Luiz Eduardo Rodrigues, Amanda Lins Alecrim-Zeza, Liliane de Araújo Ferreira, Caio dos Santos Trettel, Gabriela Mandú Gimenes, Adelson Fernandes da Silva, Celso Pereira Batista Sousa-Filho, Tamires Duarte Afonso Serdan, Adriana Cristina Levada-Pires, Elaine Hatanaka, Fernanda Teixeira Borges, Marcelo Paes de Barros, Maria Fernanda Cury-Boaventura, Gisele Lopes Bertolini, Priscila Cassolla, Gabriel Nasri Marzuca-Nassr, Kaio Fernando Vitzel, Tania Cristina Pithon-Curi, Laureane Nunes Masi, Rui Curi, Renata Gorjao, Sandro Massao Hirabara

**Affiliations:** ^1^Programa de Pós-graduação Interdisciplinar em Ciências da Saúde, Universidade Cruzeiro do Sul, São Paulo, São Paulo, Brazil; ^2^Department of Molecular Pathobiology, University of New York, New York, NY, United States; ^3^Divisão de Nefrologia, Departamento de Medicina, Universidade Federal de São Paulo, São Paulo, SP, Brazil; ^4^Department of Physiological Sciences, Biological Science Center, State University of Londrina, Londrina, PR, Brazil; ^5^Departamento de Ciencias de la Rehabilitación, Universidad de La Frontera, Temuco, Chile; ^6^School of Health Sciences, College of Health, Massey University, Auckland, New Zealand; ^7^Instituto Butantan, São Paulo, Brazil

**Keywords:** metabolism, inflammation, oxidative stress, renin-angiotensin-aldosterone system, SARS-CoV-2

## Abstract

Coronavirus disease 2019 (COVID-19) is triggered by the SARS-CoV-2, which is able to infect and cause dysfunction not only in lungs, but also in multiple organs, including central nervous system, skeletal muscle, kidneys, heart, liver, and intestine. Several metabolic disturbances are associated with cell damage or tissue injury, but the mechanisms involved are not yet fully elucidated. Some potential mechanisms involved in the COVID-19-induced tissue dysfunction are proposed, such as: (a) High expression and levels of proinflammatory cytokines, including TNF-α IL-6, IL-1β, INF-α and INF-β, increasing the systemic and tissue inflammatory state; (b) Induction of oxidative stress due to redox imbalance, resulting in cell injury or death induced by elevated production of reactive oxygen species; and (c) Deregulation of the renin-angiotensin-aldosterone system, exacerbating the inflammatory and oxidative stress responses. In this review, we discuss the main metabolic disturbances observed in different target tissues of SARS-CoV-2 and the potential mechanisms involved in these changes associated with the tissue dysfunction.

## Introduction

At the end of 2019, in Wuhan (Hubei Province, China), the burgeon of a severe acute respiratory syndrome caused by the SARS-CoV-2 coronavirus reached the condition of pandemic (COVID-19; Singhal, 2020), which caused unprecedented damage to worldwide public health and global economy. Several research groups all over the world embraced the hard task to study and understand the cellular mechanisms by which the coronavirus interacts and fuse with its target cells. The SARS-CoV-2 coronavirus enters target cells through molecular interactions of the spike protein (S protein, encoded by SARS-CoV-2 RNA), with angiotensin-converting enzyme receptor-2 (ACE-2) enzyme receptor of the target cell, precisely in the plasma membrane. The N-terminal domain of the S1 subunit (S1-NTD) can be the central point that SARS-CoV-2 uses to connect to the cell membrane in different tissues (Li, 2015). This domain creates hidden binding sites for glycans containing sialic acid. This may be important for infection and the ability of the virus to localize and interact with ACE2, the main host cell surface receptor (Robson, 2020). Currently, it is not completely understood if high blood glucose levels further increase or facilitate S1-NTD interaction to the host cell membrane, but there is some evidence that the affinity of the S protein is modulated by glycation of the ACE2 receptor, as demonstrated by *in silico* and computational analysis (Sartore et al., 2021). This modulation can shift or facilitate the virus entry into host cells by alternative pathways. When diabetic patients are early treated with insulin during COVID-19, they present a better diagnosis disease prognosis, with alleviation of the COVID-19-induced lung injury (Nakhleh and Shehadeh, 2020). Further studies will be fundamental to fully comprehend the effect of hyperglycemia on SARS-CoV-2 infection and the subsequent metabolic modulation.

Neuropilin-1 (NRP-1), expressed mainly in the respiratory and gastrointestinal tissues, can act in conjunction with ACE2 as a gateway to SARS-CoV-2 (Viana et al., 2022). After cleaved by furin, the fragment S1 containing the C-terminus (CendR) binds to the domain b1 of NRP1, contributing to the elevated virus infection process (Cantuti-Castelvetri et al., 2020). The mechanisms involved in this pathway may be associated with angiogenesis and vascular permeability (Daly et al., 2020). The high expression of NRP1 in olfactory epithelial cells can be observed in patients with COVID-19, suggesting its role in potentiating viral infection and being the target of therapies to control the disease (Cantuti-Castelvetri et al., 2020). In the initial stage of infection, SARS-CoV-2 acts mainly on lung cells. The effects on this tissue have been extensively studied and they are associated with a severe and chronic inflammatory condition, lastly leading to the “cytokine storm,” which dramatically increases the neurosusceptibility of the lung tissue. This pathological condition is commonly observed in patients with hypoxia, associated to progressive tissue injury caused by the impairment of the vascular network and peripheral nerves (Li et al., 2020b).

However, COVID-19 is not restricted to the lungs and respiratory manifestations, but also leads to dysfunction in heart, liver, kidneys, central nervous system (CNS), intestine and skeletal muscles. Therefore, the disease is better described as a multiorgan disease with multiple and/or persistent sequels, even after the disease cure. Complications derived from the SARS-CoV-2 were observed mainly in patients affected by comorbidities, such as type 2 *diabetes mellitus* (DM2), hypertension, cardiovascular diseases and, especially, older individuals (Huang et al., 2020). In this review, we describe the main molecular and cellular mechanisms involved in the SARS-CoV-2 metabolic modulation in different tissues.

## Potential mechanisms involved in metabolic modulation

### SARS-CoV-2, metabolism and inflammation

Cellular metabolism encompasses several pathways that function in synchrony to maintain cellular homeostasis and ensure adequate energy supply under different conditions. Several metabolic pathways are continuously modulated during the initiation and progression of infectious diseases, especially the intermediary metabolism pathways, e.g., glycolysis, the tricarboxylic acid cycle coupled with oxidative phosphorylation, mitochondrial and peroxisomal beta oxidation, the phosphate pentose pathway (PPP), proteogenesis/proteolysis, among others (Andrade Silva et al., 2021).

Some studies have shown that virus infections normally lead to cellular reprogramming, particularly involving glucose, amino acid/protein, and lipid metabolism in immune cells (Heaton and Randall, 2010; Vastag et al., 2011; Thai et al., 2014; Moreno-Altamirano et al., 2019; Thaker et al., 2019; Andrade Silva et al., 2021). These metabolic changes comprise viral strategies to circumvent the protective responses elicited immune cells under viral attack. However, due to the plasticity of immune cells, the initial viral infection induces an even more severe tissue inflammation, as observed in most patients infected with SARS-CoV-2 (Huang et al., 2020; Lucas et al., 2020; Andrade Silva et al., 2021). The COVID-19 affects both systemic and local homeostasis in circulating cells and infected tissues, compromising the function of various organs and, thus, aggravating symptoms and contributing to the severity of the disease (Bruzzone et al., 2020; Wu et al., 2020a; Andrade Silva et al., 2021).

COVID-19 patients regularly exhibit increased glycolytic activity in epithelial and immune cells (Codo et al., 2020; Andrade Silva et al., 2021). Monocytes infected with coronavirus also show increased ACE-2 expression and viral load dependence on glucose concentration, as well as increased glycolytic capacity and glucose reserve. These changes are not observed in monocytes infected with other viruses, such as influenza A and respiratory syncytial virus (RSV; Codo et al., 2020). The gene expression for inflammatory cytokines, including tumor necrosis factor-alpha (TNF-α), interleukin-6 (IL-6), interleukin-1beta (IL-1β), interferon-alpha and-beta (INF-α and INF-β), is dependent on the dose of glucose and viral replication. However, when the glucose uptake is blocked (by 2-deoxy-D-glucose-2-DG, for example), the expression of ACE-2 and cytokines decreases. Blocking ATP synthase, which results in an increased glycolytic pathway activity, concomitant increases in viral load are also observed, demonstrating that glycolysis is essential for viral replication in monocytes, and apparently in epithelial cells, as well (Bojkova et al., 2020; Codo et al., 2020; Andrade Silva et al., 2021).

Andrade Silva et al. (2021) suggested that this modulation in glucose metabolism in infected cells is protagonized by hypoxia-inducible factor-1 alpha (HIF-1α), which is responsible for the regulation of genes involved in the transport and processing of glucose, but also with the expression of lactate dehydrogenase-A (LDH-A), 6-phosphofructo-2-kinase/fructose-2,6-bisphosphatase-3 (PFKFB-3), glucose transporter-1 (GLUT-1) and pyruvate kinase-M2 (PK-M2). It has been shown that these genes are overexpressed in monocytes infected by SARS-CoV-2, and again, with a very distinguished metabolic pattern than other viral infections (Codo et al., 2020; Andrade Silva et al., 2021).

Diabetic patients usually present higher severity of COVID-19 symptoms and disease progression than patients with normal. This can be explained, at least in part, by the close interplay between the infection-induced glycolysis and SARS-CoV-2 replication itself (Ceriello, 2020; Codo et al., 2020). Poor glycemic control also predicts an increased need for medications and hospitalizations with proportional mortality rates (Cai et al., 2020; Liu et al., 2020a; Shauly-Aharonov et al., 2021; Yang et al., 2021b). According to this assumption, the hyperglycemia observed in DM is recognized as an independent predictor of morbidity and mortality in patients with SARS-CoV-2. In addition, hyperglycemia leads to the overproduction of reactive oxygen species (ROS), which can impose oxidative stress to the infected organism (Al-Kuraishy et al., 2021). Reactive nitrogen species (RNS) and advanced glycation end products (AGEs) are also related to this harmful stress condition (Chernyak et al., 2020). Indeed, it has been described that diabetic patients present increased risk of general infections (Shah and Hux, 2003; Casqueiro et al., 2012; Chavez-Reyes et al., 2021) and a much worse prognosis for COVID-19, with a high risk of morbidity and mortality (Lim et al., 2021; Gorjao et al., 2022).

Some studies have also reported a high number of immune cells in the infected lung, which is related to the fatality of the disease (Youngs et al., 2021). These findings support the hypothesis that a subclinical inflammatory state is one of the hallmarks of COVID-19 pathology (Rahmani-Kukia and Abbasi, 2021). Some patients also present a pathophysiological condition called “cytokine storm.” In this sense, several mechanisms have been proposed to explain why virus-induced inflammation supposedly increases insulin resistance (IR; Gangadharan et al., 2021). One of the proposed ideas indicates that the activation of inflammatory cells affects the functions of the skeletal muscle and liver, which are the main insulin-responsive organs accountable for most hormone-mediated glucose uptake (Gangadharan et al., 2021). In addition to muscle asthenia, patients with severe COVID-19 have increased liver enzyme activity, which may suggest multiple organ failure, especially during cytokine storms (Groop et al., 1989). Undoubtedly, a multitude of metabolic factors connects DM pathophysiology with the development and progression of COVID-19.

The term “cytokine storm” is used to describe the exacerbated production of proinflammatory cytokines accompanied by a weak IFN-γ response. SARS-CoV-2 can quickly activate Th1 cells, stimulating the cytokine secretion, such as IL-6 and the granulocyte-macrophage colony-stimulating factor (GM-CSF). This factor is responsible for activating CD14^+^ and CD16^+^ inflammatory monocytes to produce large amounts of IL-6, TNF-α, and other cytokines (Hu et al., 2021). Membrane-bound immune receptors, such as Fc and toll-like receptors, can contribute for a deregulated inflammatory response, and a weak induction of IFN-γ, which may additionally amplify the proinflammatory cytokine production. NETosis, a program for formation of neutrophil extracellular traps (NETs), may also contribute to the release of proinflammatory cytokines and, thus, to the “cytokine storm.” NETs comprehend extracellular nets of DNA, histones, microbicide proteins, and oxidizing enzymes, which are released by neutrophils. When the release of NETs is not properly regulated, these molecules can potentially initiate and propagate inflammatory responses and thrombosis process (Porto and Stein, 2016; Twaddell et al., 2019). Although there are no direct assessments that NETs contribute to the cytokine storm in the respiratory failure in patients with severe COVID-19, although evidence indicates that proinflammatory cytokines, such as IL-1β and IL-6, are closely linked to NET production during severe infection. In addition, during dynamic cytokine storm, T-cell lymphopenia and NETs have been associated with the severity of COVID-19 (Hu et al., 2021).

Infection and inflammation induce changes in the lipid and lipoprotein profile, leading to impaired immune responses (Stasi et al., 2021). Evidence shows that bacteria, fungi and even viruses can modulate lipid metabolism pathways, affecting lipogenesis and storage, as well as immune signaling and the efficiency of tissue repair (Khovidhunkit et al., 2004; Stasi et al., 2021). Numerous studies have shown that the predisposition to the worst forms of COVID-19 (with higher mortality rates) is related to chronic diseases, such as hypertension, cardiovascular diseases and DM (Stasi et al., 2021). Interestingly, patients infected with coronavirus were shown to have lower levels of apolipoprotein A1 (Apo-A1) and high-density lipoprotein (HDL; Stasi et al., 2021). It is known that a decrease in serum HDL is a predictive factor for the severity of COVID-19 and its worse prognosis, which is considered a risk factor that requires more attention and more intensive treatments (Stasi et al., 2021). More studies addressing the effects of dyslipidemia and lipid metabolism in COVID-19 pathology are truthfully necessary.

There are few studies that establish the relationship between metabolism and age or sex, most of which are directed to the risk of infections (Chew et al., 2021; Kautzky-Willer et al., 2022). It is important to point out that metabolic alterations are different in the acute stage of SARS-CoV-2 infection and after it, as for example during long-COVID-19 (Sykes et al., 2021). A study conducted Pasini et al. (2021), in elderly of both sexes affected by long-COVID-19, with a mean age of 72 years, showed an increase in ferritin and D-dimer concentration, as well as a reduction in hemoglobin and albumin levels. Dillard et al. (2022) conducted a study capable of showing differences in tryptophan metabolism in samples of patients affected by COVID-19 in a non-acute manner, which may be associated with a decreased inflammation. In the same study, patients with the severe COVID-19 presented increase in the histidine and ketone metabolism, which can be linked to musculoskeletal damage. However, the comparison between gender and age was not performed. Thus, further studies addressing the association between the cellular metabolic modulation and age or sex are required to completely understand the effects of these parameters during acute or chronic COVID-19.

### SARS-CoV-2 and oxidative stress

Reactive oxygen and nitrogen species (ROS/RNS) are natural products of the cellular oxidative metabolism and are also fully engaged in cellular homeostasis: from activation factors of cellular defensive systems to promoters of oxidative and nitr(osyl)ative modifications in biomolecules causing cellular dysfunction (Zarkovic, 2020). In fact, the rate of ROS/RNS production and accumulation is counteracted by the antioxidant activity *in situ*, and this pro−/antioxidant balance dictates the redox status in all cellular or extracellular compartments. Every organelle or extracellular environment has its particular redox state (or redox potential) for optimized functionality, e.g., peroxisomes and mitochondria are notably highly oxidizing compartments, whereas endoplasmic reticulum works under much more reductive conditions (Benhar, 2020). It is now understood that these intracellular redox circuits are fully integrated (also with extracellular interplays) in a redox network that performs through the diffusion of freely diffusible ROS/RNS (hydrogen peroxide and nitric oxide; H_2_O_2_ and NO•, respectively) (Sies, 2017; Chachlaki and Prevot, 2020), some sulfur-dependent metabolites, such as sulfidic acid (H_2_S; Nishida et al., 2016), or even more complex molecules, like 8-nitroguanosine 3′,5′-cyclic monophosphate (8-nitro-cGMP; Fujii and Akaike, 2013). All these redox microcircuits are real-time sensed and adjusted by several thiol (SH)-responsive proteins (cystein-dependent redox switches) e.g.Keap1-Nrf2, peroxiredoxins, thioredoxins, glutathione (Moldogazieva et al., 2018). These redox sensors are linked to transcription factors and redox-signaling cascades in order to regulate specific gene expressions that will adjust the overall redox status, cellular metabolism, protein turnover, and cell survival of that particular cell to those specific physiological conditions (Jones and Sies, 2015; Radzinski et al., 2021).

The rupture of redox homeostasis and alterations in thiol-dissulfide ratio (SH/SS) were already observed in host cells during viral infection (Li et al., 2017). Several studies have suggested that the overproduction of ROS/RNS and unbalanced cellular antioxidant-oxidant processes are determinant molecular events in the pathogenesis of respiratory viral infections, including the SARS-CoV-2 infection (Camini et al., 2017). The induced unfavorable redox condition imposed by viral infection in hosting cells, in fact, propagate towards vicinal uninfected cells by the diffusion of prooxidant agents, such as lipid hydroperoxides (LOOH) and peroxynitrite (ONOO^−^), or by triggering local inflammation processes with the release of chemotactic cytokines (Bartolini et al., 2021). As aforementioned, recent studies published during the recent pandemic period suggest that other pathologies with redox imbalance, such as diabetes, hypertension, and pulmonary, cardiac, and kidney diseases, severely increase the lethality of respiratory viral infections, mainly because of the pre-existent redox imbalances imposed by those comorbidities (Abdi et al., 2020; Lee et al., 2020).

Robust evidence has now shown that specific (SH/SS) balance is crucial for SARS-CoV-2 viral entry and fusion into hosting cells (Suhail et al., 2020). The redox conditions in hosting cells are determinant since specific thiol (-SH) groups of membrane proteins in hosting cells act as chemical anchors for molecular interactions with viral envelope glycoproteins (Lavillette et al., 2006). It has also been shown that thiol-disulfide rearrangements within viral envelopes can cause conformational changes of the S peptide, with variable affinities for viral glycoproteins and, consequently, varied fusion and engulfment capacities into a hosting cell (Fenouillet et al., 2007). Accordingly, recent studies have been exploring thiol-based chemical probes that act as reducing agents (P2119 and P2165) and antioxidants as potential inhibitors of infection by human coronaviruses, including SARS-CoV-2 (Fernandes et al., 2020; Shi et al., 2022).

ACE-2 is the host cellular receptor that locks onto the surface S protein of the highly transmissible (and deadly) SARS-CoV-2, the virus responsible for the COVID-19 pandemic (Baughn et al., 2020). ACE-2 is a membrane-bound protein and it is responsible for the hydrolysis of angiotensin II (a vasoconstrictor; Ang II) to angiotensin 1–8 (a vasodilator; Ang 1–7; Morris et al., 2020). Ang II produces ROS/RNS by stimulating membrane-bound NADPH oxidase (Wen et al., 2012). The transformation of Ang II to Ang 1–7, catalyzed by ACE-2, mitigates oxidative stress as it inhibits NADPH oxidase, and therefore, also limits ROS/RNS accumulation (Kim et al., 2012). Interestingly, the ☐-glutamyl-tripeptide glutathione (GSH) is a protagonist of the thiol-dependent antioxidant defenses in mostly all biological systems, and its reduced/oxidized ratio (GSH/GSSG) is fully dependent on NADPH levels and the Pentose Phosphate Pathway activity in cells (Perez-Torres et al., 2022). All these mechanisms shed light on the key participation of the renin-angiotensin-aldosterone system (RAAS) in the redox imbalances associated with viral respiratory diseases (Morris et al., 2020). If ACE-2 is bound to the S protein, the cellular concentration of Ang II will increase, leading to overproduction of superoxide radicals (O_2_•^−^) and other ROS/RNS that will exacerbate the oxidative conditions *in situ* and, ultimately, increasing the risk of severe illness from COVID-19.

### Renin-angiotensin-aldosterone system

The renin-angiotensin-aldosterone system (RAAS) acts mainly on the cardiovascular and renal systems, with strong links with inflammatory processes, and has both a classical and a nonclassical pathway with opposite effects (Powers et al., 2018). In the classical pathway, the angiotensinogen released by the liver is cleaved in the circulation by renin and released in the kidney to form angiotensin I; this, in turn, forms angiotensin II (Ang II) through the action of the angiotensin-converting enzyme (ACE). Ang II acts predominantly on the type 1 receptor (AT1R), resulting in physiological actions such as vasoconstriction, sympathetic activation, water and sodium retention, inflammation and fibrosis (Cabello-Verrugio et al., 2015; Hersh et al., 2022). However, Ang II can also act on the AT2R receptor and promote vasodilation, apoptosis and anti-inflammatory, anti-proliferative and antioxidative actions. In the nonclassical pathway, ACE-2 is responsible for the cleavage of Ang I (1–10) and Ang II (1–8), leading to the formation of the derivatives Ang (1–9) and Ang (1–7). The latter binds to the MAS receptor, promoting effects similar to those triggered by AT2R in addition to diuretic, natriuretic, antifibrotic and antihypertrophic effects. ACE-2 has additional affinity for other vasoactive substrates, including apelin-13 and bradykinin (Gao et al., 2020; Oz and Lorke, 2021).

ACE-2 is an enzyme bound to cell membranes in the lungs, CNS, skeletal muscle, endothelium, heart, liver, kidneys, and intestine. SARS-CoV-2 infection begins mainly through the interaction between the virus S protein and the ACE-2 receptor, which promotes proteolytic cleavage, viral entry and internalization of ACE-2. The ectodomain of ACE-2 is cleaved by the ADAM17 and the cytoplasmatic domain by the transmembrane protease serine protease-2 (TMPRSS-2), both enzymes are important for the viral infection (internalization and shedding de ACE2). The activity of ADAM17 is promoted by Ang II, leading to a reduction in the expression of ACE-2 in tissues, although high circulating levels are still present (Gonzalez et al., 2020; Bekassy et al., 2022).

SARS-CoV-2 contagion and the internalization of ACE-2 induce a decrease in the activity of ADAM17, promoting an imbalance of the renin angiotensin system (RAS) with greater activation of the ACE-2/AngII/AT1R axis, as well as reduction of the ACE-2 axis/Ang 1-7/MAS, favoring the most inflammatory, fibrotic, and apoptotic state (Gonzalez et al., 2020). In addition to the oxidative stress, which also activates the NFkB pathway, the inflammatory response is exacerbated. RAAS is present in several tissues, including skeletal muscle, which is also linked to the regulation of muscle mass and may contribute to muscle atrophy as a result of the disease (Frantz et al., 2018; Powers et al., 2018).

In addition, the RAAS interacts with other systems, such as the complement system and the kallikrein–kinin system (KKS), which also acts in the inflammatory process, contributing to the cytokine cascade, endothelial dysfunction, injury and tissue remodelling, as well as increased vascular permeability accompanied by edema and thrombosis after infection by SARS-CoV-2 (Bekassy et al., 2022). Finally, the proinflammatory process generated by the activation of these systems and direct disturbances caused by the virus will have metabolic consequences.

### Effects of SARS-CoV-2 on endocrine function, clotting, and thrombosis

Several endocrine glands, such as the pancreas, pituitary, thyroid, adrenals, tests, and ovaries, express TMPRSS2 and ACE2, two main receptors for the SARS-CoV-2 virus, suggesting that the virus can infect and modulate the function of these endocrine glands (Kazakou et al., 2021). It has been observed that the SARS-CoV-2 infection impairs glucose homeostasis (Kazakou et al., 2021) and increased blood glucose level is found in COVID-19 patients who were admitted and hospitalized, including patients with or without diabetes, which can worsen the prognosis, severity of the disease, and mortality from these patients (Bode et al., 2020; Kim et al., 2020; Wu et al., 2020b; Wang et al., 2020c; Kazakou et al., 2021; Lazarus et al., 2021). In addition, inflammation caused by the virus infection can induce insulin resistance, contributing to the hyperglycemia associated with counterregulatory hormonal dysregulation (Dungan et al., 2009; Kazakou et al., 2021). Similarly to the effects observed in other respiratory syndromes, activation of inflammatory cells during SARS-CoV-2 infection can modulate the function of liver and skeletal muscle, two important tissues involved in glucose homeostasis, which can result in hyperinsulinemia and hyperglycemia (Channappanavar and Perlman, 2017; Kazakou et al., 2021). Additionally, prolonged hospitalization of critically ill patients results in physical inactivity, muscle loss and weakness, contributing to decreased insulin response, especially in patients who survive after sepsis and acute respiratory distress syndrome (ARDS; Rocheteau et al., 2015; Pfoh et al., 2016; Kazakou et al., 2021). Cases of rhabdomyolysis during viral infection have been also reported, which may exacerbate glycolytic dysregulation (Jin and Tong, 2020; Kazakou et al., 2021). Evidence showing that SARS-CoV-2 infection is associated to death of pancreatic β cell death, deficiency of insulin release, and consequently hyperglycemia has been found (Hollstein et al., 2020; Kazakou et al., 2021).

Increased production of chemokines and cytokines by the immune system during COVID-19 also can harm pancreatic β cell function, instilling insulin-glucose-dependent dose sensitivity (Rubino et al., 2020; Li et al., 2020a; Kazakou et al., 2021). The cellular damage of pancreatic islets is facilitated by the increased expression of ACE2 by these cells during infection (Letko et al., 2020; Liu et al., 2020b; Kazakou et al., 2021; Akarsu et al., 2022). This increase is retated to the release of inflammatory cytokines, cell death by apoptosis of β cells, and consequently decreased insulin secretion (Yang et al., 2020; Kazakou et al., 2021). Culture of pancreatic islet cells from post-mortem biopsies has shown that, after SARS-CoV-2 infection, several changes occur in these cells, including altered morphology, transcription, and function, and decreased hormone secretory granules, mainly compromising insulin release (Kazakou et al., 2021; Muller et al., 2021; Tang et al., 2021; Wu et al., 2021). In diabetic patients, the decrease in insulin secretion is exacerbated due to the reduction in ACE2 expression, which leads to decreased angiotensin II degradation, increased aldosterone, and renal hypokalemia (Pal and Bhansali, 2020; Kazakou et al., 2021).

Both hypothalamus and pituitary gland express ACE2, thus these tissues are potential targets for SARS-CoV-2 and it is known that about 40% of respiratory syndrome survivors had mild secondary hypocortisolism and 5% central hypothyroidism (Chiloiro et al., 2019; Kazakou et al., 2021). About 20–50% of hospitalized patients present hyponatremia. These effects can be related to the syndrome of inappropriate antidiuretic hormone secretion, due to the high levels of interleukins that can induce vasopressin release (Berni et al., 2020; Kazakou et al., 2021).

Hospitalized COVID-19 patients have to be routinely evaluated in relation to the coagulation profile, including D-dimer, thromboplastin, platelets, fibrinogen, and thromboplastin, since they present increased risk to clotting and thrombosis. This risk is elevated between 7 and 11 days after the appearance of the first symptoms or 4 and 10 days after hospitalization (Connors and Levy, 2020; Thachil et al., 2020; Gomez-Mesa et al., 2021). Approximately half of patients admitted to hospitals present changes in coagulation parameters, including elevated D-dimer, prolonged prothrombin time, low fibrinogen levels, and/or thrombocytopenia, which are associated with elevated risk for episodes of thrombosis than hemorrhagic (Gomez-Mesa et al., 2021).

The combination of prolonged prothrombin time, thrombocytopenia, and elevated D-dimer is indicative of disseminated intravascular coagulation (DIC). Coagulopathy in COVID-19 patients occurs through the combination of DIC and pulmonary thrombotic microangiopathy, which can have major impacts on organ dysfunction in most patients with the severe form of the disease (Thachil et al., 2020; Gomez-Mesa et al., 2021). In the lung microvasculature, there is a large deposit of thrombin and fibrin, which can contribute to respiratory distress syndrome and coagulopathy, especially in patients who die. Additionally, the occurrence of hypoxia can worsen thrombosis, not only by increasing blood viscosity, but also by activating the HIF-dependent signaling pathway (Tang et al., 2020; Wang et al., 2020b; Gomez-Mesa et al., 2021).

As observed in coagulopathy during sepsis, the endotheliopathy during SARS-CoV-2 infection seems to contribute to the pathophysiological changes in the microcirculation. The ACE2 receptor can cause inflammatory cell infiltration, endothelial apoptosis, and prothrombotic pathway activation (Connors and Levy, 2020; Tang et al., 2020; Gomez-Mesa et al., 2021). Other relevant coagulation abnormalities include decreased fibrinogen, increased lactate dehydrogenase (LDH) activity, and high serum ferritin, the latter observed in only a few hospitalized patients (Gomez-Mesa et al., 2021). Other acute phase reagents present in COVID-19 patients (e.g., factor VIII, Von Willebrand Factor, and fibrinogen) are associated with increased thrombosis risk. In critically ill patients, the increase in inflammatory cytokines (e.g., IL-6) can induce tissue factor expression in macrophages, generating thrombin and activating clotting. Meanwhile, IL-1 seems to suppress the endogenous coagulation cascade (Marietta et al., 2020; Thachil et al., 2020; Gomez-Mesa et al., 2021).

## Metabolic changes induced by SARS-CoV-2 in different tissues

### Changes in the central nervous system

The global increase in the number of cases of individuals infected with SARS-CoV-2 showed how this virus also substantially affects the CNS (Abdullahi et al., 2020). Some of the neurological manifestations include headache, dizziness, acute cerebrovascular disease, ataxia, seizures, loss of taste, vision problems, neuromuscular pain and impaired consciousness, which occur concomitantly or even before the onset of respiratory symptoms (Iadecola et al., 2020). The spectrum of neurological diseases associated with SARS-CoV-2 infection is also evident, including acute disseminated encephalomyelitis (Parsons et al., 2021), meningoencephalitis (Bernard-Valnet et al., 2020), encephalitis (Pilotto et al., 2020), Guillain-Barré Syndrome (Alberti et al., 2020), and encephalopathies (Garg et al., 2021).

Harapan and Yoo (2021) propose that the neurotropic mechanisms by which COVID-19 leads to neuropathologies can be based on ACE2 receptor expression. This receptor is expressed in neurons and several glial cells of the brain, including astrocytes, oligodendrocytes, black substance, ventricles, medium temporal gyre, and olfactory bulb. For example, damage in the olfactory epithelium, which express ACE2 and TMPRSS2, becomes vulnerable to SARS-CoV-2 infection, which can lead to the anosmia and other related disorders in odor perception, as frequently observed in COVID-19 diagnosed patients (Brann et al., 2020).

Among patients with a positive diagnosis for COVID-19, 37% of those admitted to the ward (Mao et al., 2020) and 84% of those admitted to the intensive care unit (ICU) (Helms et al., 2020) displayed neurological symptoms, and these were not restricted to the severe form of the disease. Markers of axonal damage, such as neurofilament light chain protein, and activation of astrocytes, including glial fibrillary acidic protein (GFAP), have been found to be increased in the serum of patients infected with SARS-CoV-2, both in mild to moderate (Ameres et al., 2020) and moderate to severe cases (Kanberg et al., 2020). At least three forms of access to the CNS by SARS-CoV-2 are possible: (1) through the bloodstream, where the virus infects endothelial cells, it is internalized in the nerve terminals by endocytosis, and transported (in a retrograde manner) and disseminated to other regions of the brain (Abdullahi et al., 2020); (2) *via* axonal transport of the olfactory tract (Butowt and Bilinska, 2020); and (3) through the infiltration of infected immune cells not resident in the CNS (Butowt and Bilinska, 2020). Once in brain tissues, the S protein present in SARS-CoV-2 interacts with ACE-2 receptors present in the membrane of neurons and glial cells, initiating infection and replication in these cells (MadaniNeishaboori et al., 2020).

Although these possibilities of brain invasion by the SARS-CoV-2 virus exist, the presence of viral genetic material in the cerebrospinal fluid (CSF) of infected patients with neurological manifestations was not regularly confirmed (Matschke et al., 2020). In addition, a histopathological study of the brain tissue of deceased COVID-19 patients barely detected RNA or viral protein in those brain samples (Lee et al., 2021). In contrast, recent findings actually demonstrated the presence of immune activation and inflammatory processes in the CSF and brain tissue related to neurological symptoms in acute COVID-19 (Song et al., 2021).

Specific structural and metabolic patterns in the brain of infected patients have been studied by magnetic resonance imaging (MRI) and functional imaging evaluations using positron emission computed tomography^18^ fluorodeoxyglucose (FDG-PET/CT) (Chougar et al., 2020; Kas et al., 2022). The local consumption of glucose, which is proportional to the FDG uptake by brain tissues, strongly correlates with local synaptic and neuronal activity (Berti et al., 2014).

A study with four older patients (+60 y) with COVID-19-related encephalopathy and, acute cognitive dysfunction (but no virus presence and/or abnormalities in the CSF), demonstrated a similar (image) metabolic pattern of prefrontal and orbital-frontal hypometabolism, with cerebellar hypermetabolism, detected by FDG-PET/CT analyses^18^ (Delorme et al., 2020). These results differ from those in patients with delirium, whom showed global cortical hypometabolism (Haggstrom et al., 2017). In encephalitis, cortical hypometabolism may be a consequence of various neuropathological mechanisms, such as direct blocking of receptors or ion channels by antibodies, structural pathogenic damage or post infection recovery mechanisms (Delorme et al., 2020). In contrast, cerebellar hypermetabolism has been reported in paraneoplastic cerebellar degeneration (Choi et al., 2006). The increase in FDG uptake may represent compensatory mechanisms (Ritz et al., 2019), electroconvulsive phenomena or inflammatory processes (of infectious or immune origin) that increase the energy requirement of affected cells (Delorme et al., 2020).

Similarly, the evaluation of 15 young COVID-19 patients (+ 18 y), with two or more neurological symptoms in the subacute stage of the disease, showed predominant frontoparietal hypometabolism (Hosp et al., 2021). The analysis of seven COVID-19 patients with acute encephalopathy also identified hypometabolism in the prefrontal cortex, anterior cingulate, insula and caudate nucleus regions, which persisted mildly or severely 6 months after the disease onset (Kas et al., 2022). Dysfunctions in the insular region may be related to the failure of respiratory perception in patients with COVID-19 (Nouri-Vaskeh et al., 2020). Symptoms of anxiety and depression were also observed concomitantly with hypometabolism in the cerebral insular region in patients with posttraumatic stress (Guedj et al., 2021).

**Figure 1:**
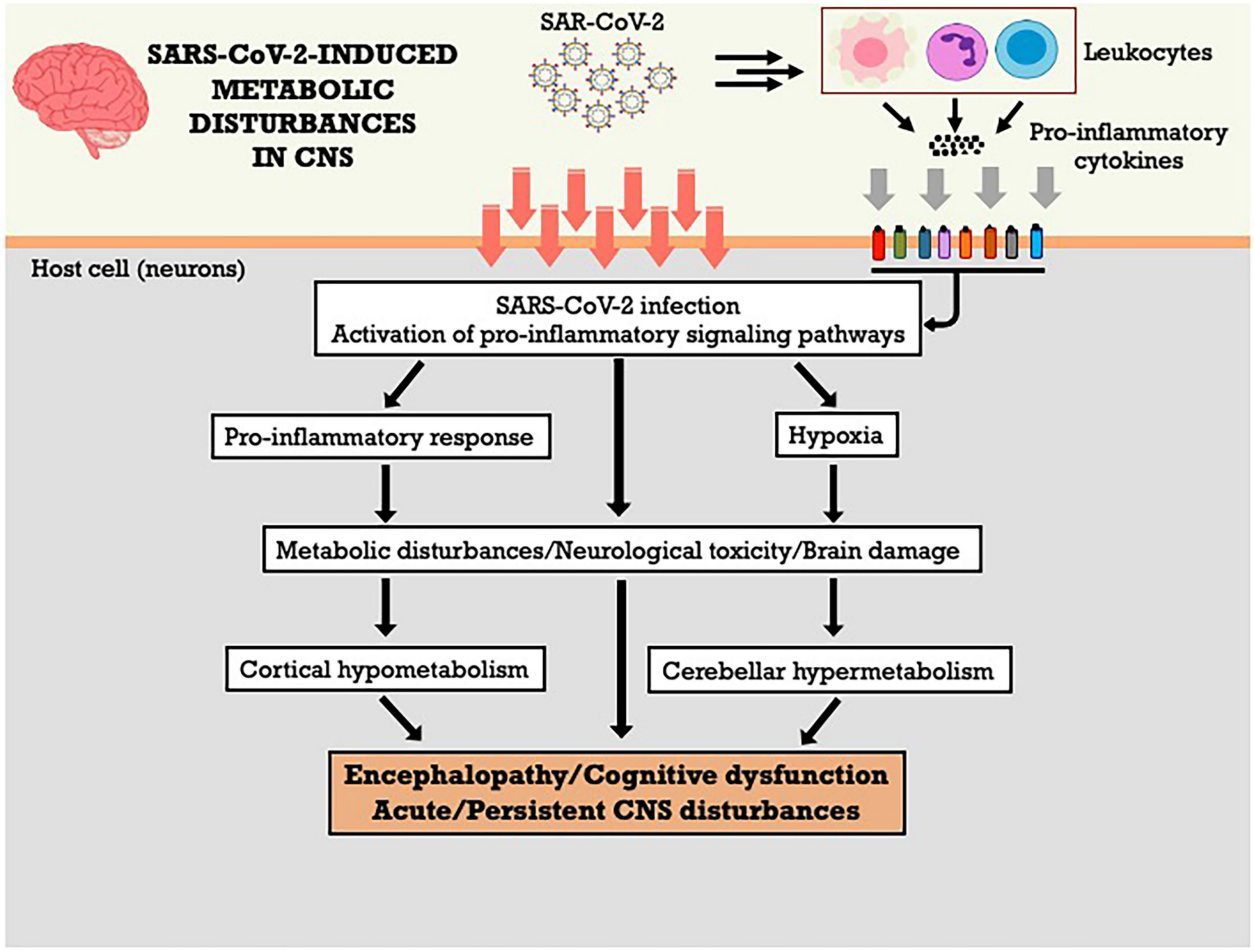
SARS-CoV-2-induced metabolic disturbances in central nervous system. CNS, central nervous system.

In cases of severe COVID-19, cognitive dysfunctions were associated to other factors, including hypoxia encephalopathy, metabolic disorders, and/or sedation side effects in the case of patients admitted to the ICU (Espindola et al., 2021). The diagnosis of encephalitis or encephalopathy related to COVID-19 can be confused with the acute delirium underlying cognitive deficits or epilepsy (Kas et al., 2022). Thus, it is necessary to analyze brain metabolism in COVID-19 patients with acute CNS dysfunction, in order to determine the pathophysiological basis of such peculiar cortical hypometabolism and cerebellar hypermetabolism, aiming to elucidate whether these abnormalities are due to transient or irreversible brain damage. The Figure 1 summarizes the metabolic effects of SARS-CoV-2 on CNS and the potential mechanisms involved.

### Modulation in skeletal muscle

The skeletal muscle tissue expresses ACE-2 and is potentially vulnerable to SARS-CoV-2 and direct myotoxicity from the infection (Ferrandi et al., 2020; Li et al., 2020b). However, some studies did not find immunohistochemical evidence of viral infiltration in skeletal muscle obtained from autopsy, despite histological evidence of muscle inflammation and damage (Aschman et al., 2021; Suh et al., 2021). Nevertheless, the skeletal muscle of COVID-19 patients may be affected by other aspects of the disease, suffering from muscle atrophy, weakness and reduced tolerance to exercise (fatigue), which are amongst the most common symptoms of acute and “long COVID” (Post-Acute Sequelae of SARS CoV-2 infection – PASC; Nasiri et al., 2020; Aiyegbusi et al., 2021; Ali and Kunugi, 2021; Han et al., 2022; Montes-Ibarra et al., 2022; Rao et al., 2022; Wang et al., 2022).

The systemic inflammation elicited by COVID-19, increases the levels of several inflammatory molecules, including C-reactive protein, IL-1β, IL-2, IL-6, IL-8, IL-10, and TNF-α (Tay et al., 2020; Mulchandani et al., 2021). The specific effects of this COVID-induced cytokine storm on skeletal muscle are yet to be elucidated, but it is known that sustained inflammation can promote insulin resistance, reduce protein synthesis and increase proteolysis, leading to muscle loss (Kanova and Kohout, 2022). COVID-associated physical inactivity and muscle disuse due to bed rest or quarantine, low nutrient intake, and nutrient loss by vomiting and diarrhea could also be factors intensifying muscle atrophy (Ali and Kunugi, 2021; Montes-Ibarra et al., 2022; Soares et al., 2022). Moreover, exacerbated or prolonged exposure to proinflammatory cytokines may impair proliferation and differentiation of satellite cells, while stimulating deposition of extracellular matrix, reducing regenerative capacity and causing fibrosis (Disser et al., 2020). Inflammation can also promote oxidative stress and mitochondrial dysfunction, reducing the oxidative capacity of muscle fibers (Kanova and Kohout, 2022). Indeed, a histopathological analysis of the biceps brachii muscle of participants recovered from COVID-19 but experiencing fatigue/weakness or myalgia for at least 3 months, showed signs consistent with inflammation, fiber atrophy, fibrillar damage, altered mitochondrial structure, and vascular degeneration (Hejbol et al., 2022).

Blood vessels are affected by inflammation, oxidative stress and RAAS imbalance, resulting in endothelial injury and dysfunction, and formation of microthrombi (Jin et al., 2020). Ratchford et al. (2021) showed altered vascular function in healthy young adults 3–4 weeks after testing positive for COVID-19, with a reduction in femoral artery blood flow response and brachial artery flow-mediated dilation, and an increase in carotid artery stiffness. Such deterioration of vascular function can lead to skeletal muscle hypoperfusion and hypoxia, potentially increasing muscle wasting and altering substrate utilization in the tissue, with increased reliance on glycolytic metabolism (Soares et al., 2022). Peripheral nerve damage by viral infection and inflammation may also negatively impact motoneuron function, contributing to muscle atrophy and weakness (Vanhorebeek et al., 2020; Soares et al., 2022). Those effects are well-described for critical illness myopathy (Shepherd et al., 2017; Vanhorebeek et al., 2020) but it remains to be determined if the same occurs with COVID-19 patients.

**Figure 2:**
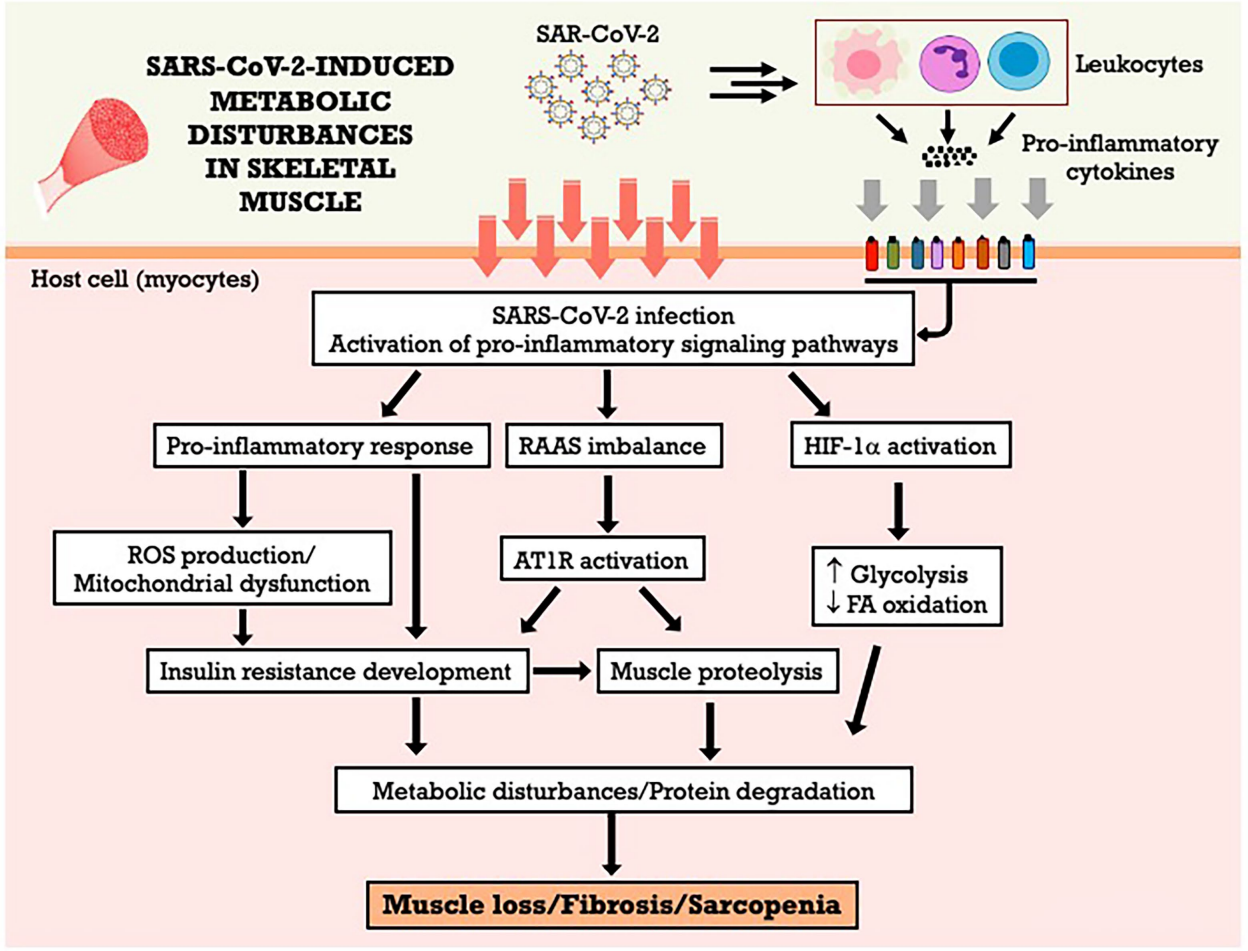
SARS-CoV-2-induced metabolic disturbances in skeletal muscle. AT1R, angiotensin II type 1 receptor; FA, fatty acid; HIF-1α, hypoxia-inducible factor-1 alpha; RAAS, renin-angiotensin-aldosterone system; ROS, reactive oxygen species.

The RAAS imbalance during COVID-19, favoring the activation of ACE/AngII/AT1R axis in the expense of the ACE-2/Ang (1–7)/MAS axis (Gonzalez et al., 2020) is likely to have direct effects the occurrence of skeletal muscle anabolic resistance and muscle atrophy. Ang II signal transduction promotes serine phosphorylation of the insulin-1 receptor substrate (IRS-1), decreasing the activation of the IGF1/Akt/mTOR pathway, one of the main anabolic pathways controlling protein synthesis and degradation in muscle (Frantz et al., 2018). Additionally, AngII and AT1R activate inflammatory pathways and induce ROS formation, contributing to insulin resistance, protein degradation and skeletal muscle loss and fibrosis (Cabello-Verrugio et al., 2015; Delafontaine and Yoshida, 2016; Frantz et al., 2018; Gonzalez et al., 2020). The ACE-2/Ang 1-7/MAS axis, in an antagonistic way, could directly inhibit AT1R activation, improving insulin signaling and glucose uptake (Cabello-Verrugio et al., 2015; Powers et al., 2018). However, the ACE-2/Ang (1–7)/MAS axis is suppressed during COVID-19 (Gonzalez et al., 2020), and its activation may become a potential therapeutic strategy to patients affected by the disease (Shete, 2020). The Figure 2 summarizes the metabolic effects of SARS-CoV-2 on skeletal muscle and the potential mechanisms involved.

### Kidney changes

The kidneys have a high content of ACE-2 receptors, which are expressed in several renal cells, such as the proximal tubules and podocytes, at even higher numbers than the lung cells, making the kidneys one of the main targets for SARS-CoV-2 (Ye et al., 2006; Hassanein et al., 2020; Vaduganathan et al., 2020). Particles of the virus were detected in the tubular epithelium and podocytes in autopsies of patients with the disease (Hassanein et al., 2020; Su et al., 2020).

Changes in systemic metabolism, as well as renal cell metabolism, are reported as crucial events in the decline of renal function (Andrade Silva et al., 2021). The kidneys of patients with COVID-19 show tubular lesions and necrosis, loss of brush border and decreased megalin expression. The decrease in this protein may hinder the catabolism of renal HDL, which may influence the plasma concentrations of HDL and Apo-A1 (Smith, 2020).

Infection of the renal tissue can trigger increased proteinuria, hematuria and, in more severe cases, acute kidney injury (AKI), considerably increasing the mortality and morbidity rate of these patients, especially those requiring ICU admission (Braun et al., 2020; Cummings et al., 2020; Hirsch et al., 2020; Andrade Silva et al., 2021). The effects of COVID-19 on the kidneys are still not completely clear, and its pathogenesis is multifactorial and may involve mechanisms such as interruption of RAAS homeostasis, hemodynamic instability, cytokine storms and viral cytopathic damage through ACE-2 (Stasi et al., 2021).

The occurrence of AKI in patients with COVID-19, according to the criteria of the Global Guidelines for Improvement of Kidney Diseases (KDIGO), is 36% (Stasi et al., 2021). A study with 701 patients showed that 43.9% had proteinuria, 26.7% hematuria and 13% had altered renal function (Cheng et al., 2020). In the most severe cases of the disease, kidney damage requires dialysis, and when this happens, the incidence of mortality increases considerably. In addition, patients with AKI triggered by COVID-19, even after discharge, have persistent kidney injury (Stasi et al., 2021).

There are reports of COVID-19 patients with renal failure (Zhou et al., 2020), which may be associated with an alteration in the expression of ACE-2 during the initial phases of the disease. For example, when the conversion of Ang II into Ang (1–7) by ACE-2 becomes impaired by the viral infection, high levels of Ang II accumulate and are associated with persistent vasoconstriction, heart disease, apoptosis and oxidative processes that contribute to renal failure and accelerate the progression of the disease (Soler et al., 2013; Kai and Kai, 2020; Stasi et al., 2021).

In addition, infection by SARS-CoV-2 increases glycolytic activity and the expression of inflammatory genes such as TNF-α, IL-6, IL-1β, INF-α and INF-β (Andrade Silva et al., 2021), and this inflammatory response is potentially harmful to renal tissue (de Rivero Vaccari et al., 2020). IL-6 is increased in several animal models of AKI, leading to endothelial cell activation and dysfunction, IL-8 production, neutrophil recruitment and increased endothelial permeability (Faubel and Edelstein, 2016; Singbartl et al., 2019). Additionally, it is already known that IL-6 may be a possible biomarker for the early diagnosis and development of AKI (Su et al., 2017; Stasi et al., 2021). Additionally, TNF-α and its receptor are positively regulated in AKI in ischemia and reperfusion (IR) models, causing proinflammatory changes in several remote tissues in addition to endothelial dysfunction and apoptosis of endothelial cells (Hoke et al., 2007; Faubel and Edelstein, 2016). Some *in vitro* studies suggest that tubular cells also generate inflammatory cytokines during AKI (Bijuklic et al., 2007).

Another issue that has been studied is the interaction between inflammation and oxidative stress (Saleh et al., 2020). TNF-α induces an increase in mitochondrial ROS, also modulated by IL-6, which impairs the activity of the electron transport chain and further stimulates the production of proinflammatory cytokines (Li et al., 2013; Saleh et al., 2020). The cytokines present in patients during coronavirus infection may prevent oxidative phosphorylation and ATP in cellular mitochondria, which could cause membrane permeabilization, changes in mitochondrial dynamics, ROS production, and cell death by apoptosis (Naik and Dixit, 2011; Jo et al., 2016; Saleh et al., 2020).

ROS are elevated in the mitochondria of the injured kidney, even with decreased oxygen consumption, as observed by González-Flecha and Bovertis (Gonzalez-Flecha and Boveris, 1995). The production of ROS, in turn, is accompanied by increased NFκB pathway activation, imbalance of cytoplasmatica calcium levels, and release of mitochondrial DNA into the cytosol, which can lead to the production of inflammatory cytokines, such as IL-1β, activating the inflammasome/NLRP3 and inducing the production of IL-6 (Naik and Dixit, 2011; Nakahira et al., 2011; West et al., 2015; Jo et al., 2016; Kozlov et al., 2017). These data suggest that both the inflammation can lead to oxidative stress and vice versa. In addition to apoptosis and unbalanced calcium concentrations, lipid peroxidation is also a result of excess ROS (Aksu et al., 2011).

During AKI, the kidneys can exposed to the hypoxia due to vascular damage, heart failure, pneumonia, RAAS imbalance, endothelial inflammation, thrombosis, and/or hipercoagulation. The proximal tubules are the cells most susceptible to mitochondrial dysfunction, since these cells depend on oxidative metabolism and their mitochondria are in a more oxidative state than the cells of the distal tubule, which can use the glycolytic pathway (Venkatachalam et al., 2010; Zuk and Bonventre, 2016). These damaged tubules may contribute to the inflammatory state by releasing proinflammatory cytokines, such as TNF-α, IL-1β, IL-6, IL-8, TGF-β and MCP-1, while leukocytes active in this outcome produce IL-1β, IL-6, IL-8, MCP-1 and ROS, exacerbating inflammation in a positive feedback cycle and increasing the lesion and inflammation in already compromised renal tissue (Bonventre and Zuk, 2004; Basile et al., 2012), which can worsen the state of the patient infected by coronavirus. Oxidative stress limits the glycolytic capacity in tubular cells during injury, making these cells unable to sustain normal production of ATP (Bastin et al., 1987; Uchida and Endou, 1988; Basile et al., 2012). It is noteworthy that both ATP depletion and cell damage can be reversible, as long as there is reestablishment of the energy substrate and the machinery for ATP generation is not compromised (Basile et al., 2012).

In addition, COVID-19 may increase the expression of hypoxia-inducible factor-1 (HIF-1α), a nuclear protein first seen in cells cultured in oxygen-deprived environments (Semenza and Wang, 1992; Wang and Zhang, 2020). Patients with COVID-19 may have respiratory failure, which may result in hypoxemia and, consequently, peripheral tissue ischemia, explaining the increase in HIF-1α. Additionally, the impact of hypoxia on this tissue can lead to lipotoxicity, contributing to kidney damage in patients affected by this disease (Ruidera et al., 1988; Bobulescu et al., 2008; Andrade Silva et al., 2021). Studies show that HIF-1α is renoprotective during the progression and repair phases of AKI, reducing apoptosis and necrosis of tubular cells (Hill et al., 2008; Wang and Zhang, 2020). Since acute tubular necrosis is one of the most common changes observed in patients with AKI triggered by COVID-19 (Sharma et al., 2020; Su et al., 2020; Stasi et al., 2021), the increase in HIF-1α may be protective against the nephrological damage caused by the disease. In addition, another protective mechanism of HIF-1α is to improve the cellular microenvironment, smoothing the infiltration of macrophages and inflammatory mediators (Wang and Zhang, 2020), which are evident during SARS-CoV-2 infection (Andrade Silva et al., 2021; Stasi et al., 2021).

**Figure 3:**
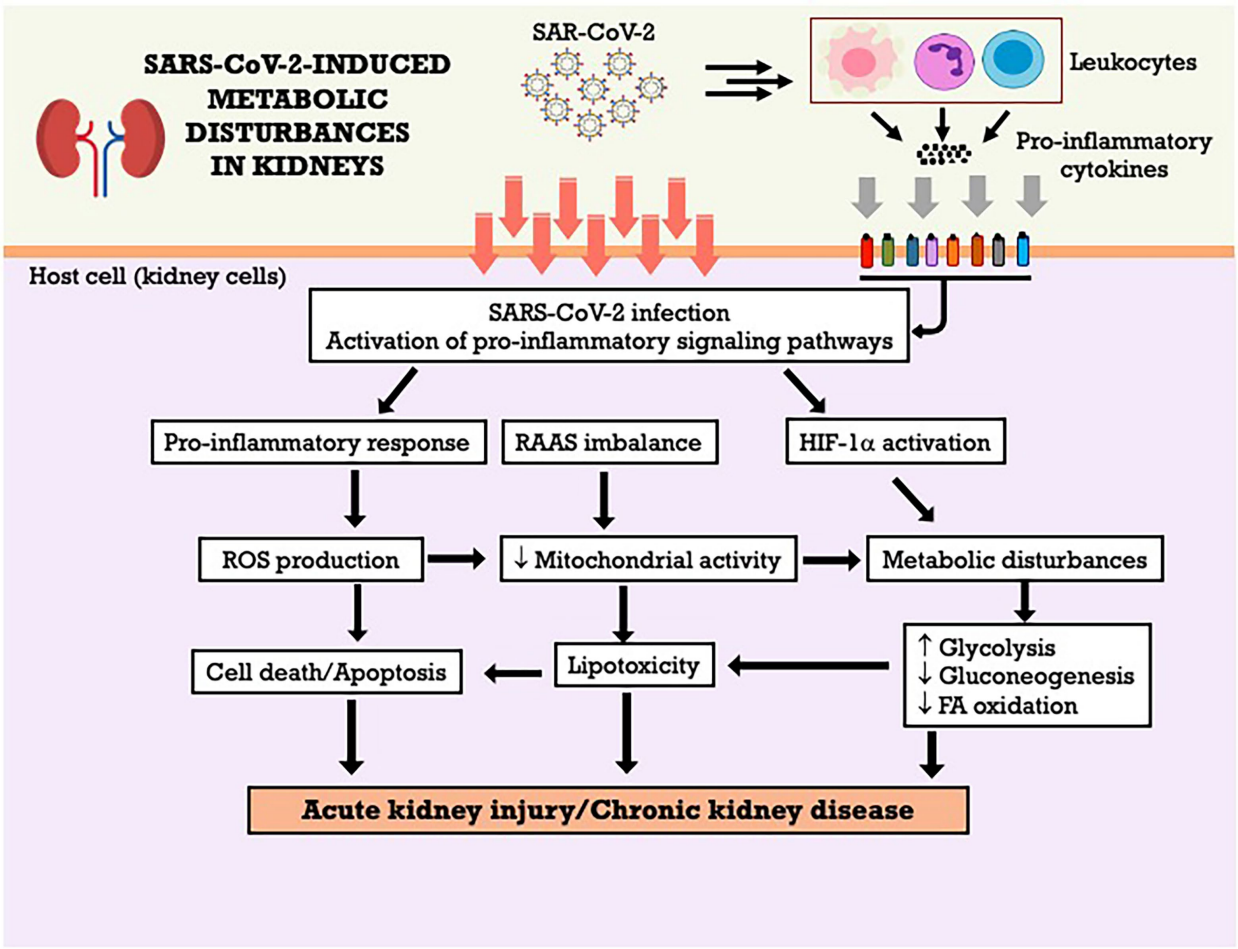
SARS-CoV-2-induced metabolic disturbances in kidneys. FA, fatty acid; HIF-1α, hypoxia-inducible factor-1 alpha; RAAS, renin-angiotensin-aldosterone system; ROS, reactive oxygen species.

It was observed that HIF-1α can also influence the function of mitochondria and regulate the oxidation of mitochondrial fatty acids (Wang and Zhang, 2020), in addition to regulating glucose metabolism (Eckardt et al., 2005). In general, HIF-1α mediates the transition from oxidative to glycolytic metabolism, thus generating ATP independently from oxygen and the conversion of the cytochrome C oxidase subunit to improve the efficiency of electron transfer during hypoxia (Fukuda et al., 2007; Shu et al., 2019). The Figure 3 summarizes the metabolic effects of SARS-CoV-2 on kidneys and the potential mechanisms involved. However, the precise mechanisms involved in metabolic dysfunction in COVID-19 are still scarce in the literature, especially when related to renal metabolism, which makes future studies and discussions necessary to elucidate this issue (Andrade Silva et al., 2021).

### Metabolic changes induced in cardiac tissue

It has been well demonstrated that patients with COVID-19 may present several cardiovascular manifestations, including chest discomfort and palpitation as initial symptoms. As described above, ACE-2 is an important functional receptor for the input of SARS-CoV-2 and is highly expressed in the heart. The susceptibility of the human heart to SARS-CoV-2 infection was evidenced by Chen et al. (2020), who showed the consistent persistence of ACE-2 in cardiac tissue, with even higher levels of expression than in the lung. ACE-2 plays an important role in the regulation of homeostasis of the cardiovascular system. The expression of high levels of ACE-2 in the heart makes it attractive for viral infection. RNA-seq analyses showed that more than 7.5% of human cardiac cells are positive for ACE-2. Ang II has the function of increasing sympathetic activity, reabsorbing electrolytes, retaining water, and inducing vasoconstriction to increase blood pressure (Ashraf et al., 2021).

The severity of COVID-19 in patients with preexisting cardiovascular diseases is higher than that in healthy individuals and it is associated with increased secretion of ACE-2 inhibitors (Zheng et al., 2020; Liu et al., 2021). According to Liu et al. (2021), the incidence of myocardial injury (MI) can vary from 7.2 to 19.7% in patients with COVID-19, with high serum levels of cardiac biomarkers or abnormalities in echocardiograms and electrocardiograms. Conversely, Shi et al. (2020) demonstrated that the association between cardiac injury and mortality is statistically significant and that cardiac injury is a common complication, comprising approximately 19.7% of cases, and it is associated with a high risk of mortality during hospitalization. In addition to the cytokine storm involving the activation and proliferation of lymphocytes and macrophages, an increase in systemic inflammatory response markers, such as C-reactive protein and procalcitonin, was also observed in patients with cardiac injury in this study. Increased release and activation of these markers may lead to apoptosis or necrosis of myocardial cells (Guan et al., 2020; Huang et al., 2020; Liu et al., 2021).

Studies have shown that inflammatory responses mediated by COVID-19 infection are divided into two categories: primary and secondary. The primary response begins after viral infection, and the secondary response begins with adaptive immunity and neutralization by antibodies. Myocardial damage is aggravated in patients with increased inflammatory activity, platelet activation and increased thromboxane synthesis. In addition to the increase in cellular inflammation induced by imbalance in the Th1 (T-helper 1) and Th2 responses, the levels of some inflammatory mediators, such as IL-10, IL-4 and IL-6, are elevated in tissue samples (Tajbakhsh et al., 2021). This cytokine storm seems to be involved in the effects observed in the cardiovascular system, including damage to the myocardium and greater disease severity. Patients with severe COVID-19 infection show increased concentrations of IL-1β, IFN-γ, TNF-α, monocyte chemotactic protein-1 and granulocyte colony stimulating factor (G-CSF).

In addition to the high expression of ACE-2, Shao et al. (2021) proposed that endothelial cells (ECs) express four types of coronavirus receptors already identified, such as membrane alanylaminopeptidase (ANPEP, CD13, a receptor for human coronavirus), cell adhesion molecule of the carcinoemory antigen family 1 (CEACAM1), dipeptidyl peptidase-4 (DPP4) and cellular serine protease 2 (TMPRSS2). These four types of coronavirus receptors are expressed in ECs in the human heart. SARS-CoV-2 preferentially uses the ACE-2 receptor to enter host cells and uses TMPRSS2 to initiate the S protein. In addition, the four types of coronavirus receptors were also expressed in the aortic ECs of mice. It was also observed that SARS-CoV-2 can use cathepsin B (CTSB) and cathepsin L (CTSL) to enter TMPRSS2-negative cells, meaning that CTSB and CTSL can replace TMPRSS2 in their function (Shao et al., 2021).

A study by Yang et al. (2021a) suggests that cardiomyocytes have high susceptibility to SARS-CoV-2 and this may be one of the cellular mechanisms of cardiac injury in patients with COVID-19. In this study, the RNA expression of ACE-2, CTSB and CTSL in the human embryonic heart at unicellular resolution was analyzed. The results suggest that atrial and ventricular cardiomyocytes are potentially susceptible to SARS-CoV-2 and that the virus can enter ventricular cardiomyocytes using CTSB/CTSL. It was also observed that differentially expressed and positively regulated genes were associated with the regulation of cardiac muscle contraction, transmembrane transport, positive regulation of ATPase activity, mitochondrial respiratory chain complex I, mitochondrial respiratory chain III and NADH dehydrogenase (ubiquinone). Conversely, differentially expressed and negatively regulated genes were also observed in the organization of the extracellular matrix, generation of extracellular exosomes and binding to structural constituent proteins of the cytoskeleton (Yang et al., 2021a).

**Figure 4:**
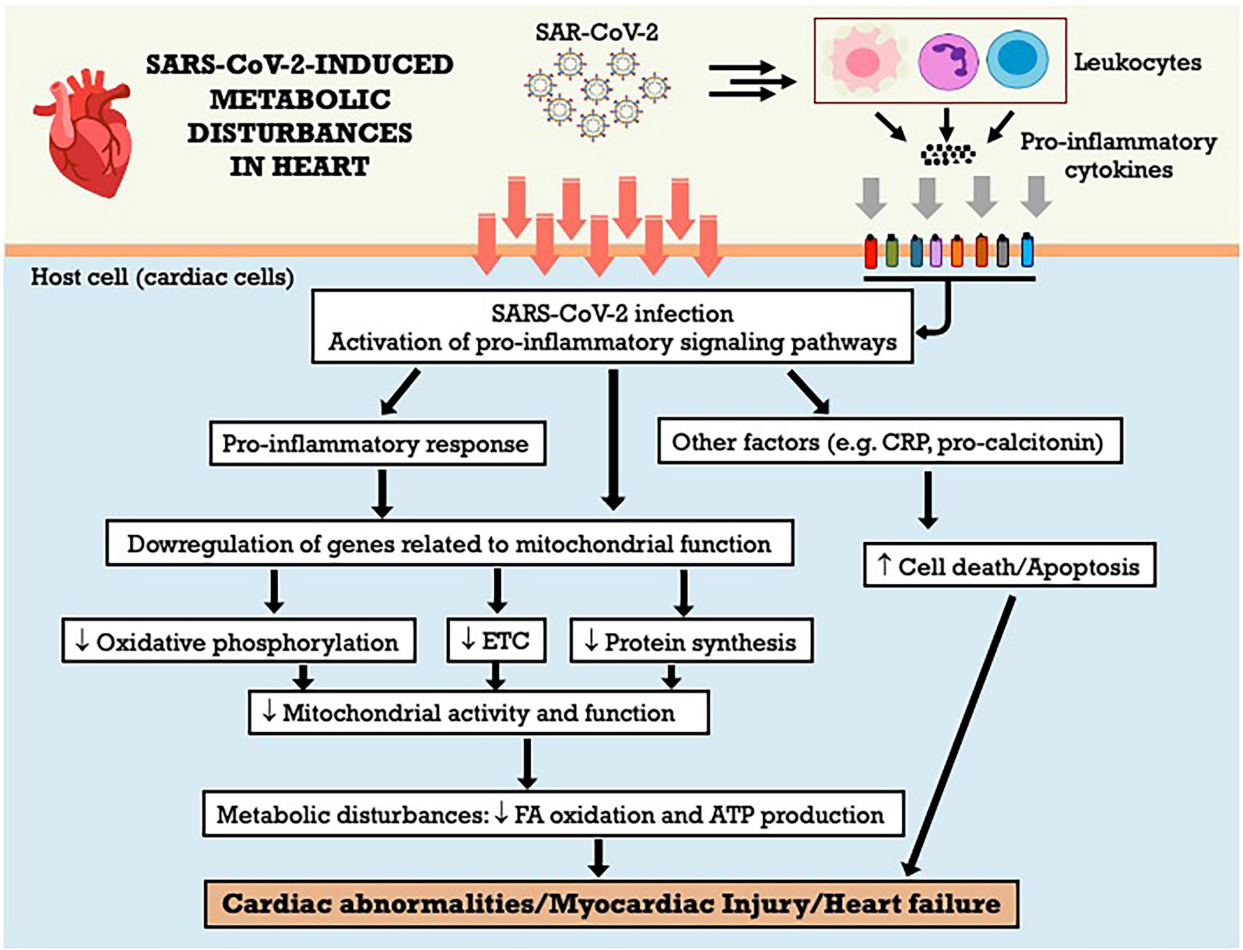
SARS-CoV-2-induced metabolic disturbances in heart. ATP adenosine triphosphate; CRP, C-reactive protein; ETC, electron transport chain; FA, fatty acid.

Studies have shown that SARS-CoV-2 infection alters mitochondrial morphology in multiple organs and cell lines of humans, especially cardiac tissue (Guarnieri et al., 2022). The analysis of transcriptional changes related to mitochondrial bioenergetics, particularly mitochondrial oxidative phosphorylation (OXPHOS) and protein synthesis genes reveals the downregulation of virtually all OXPHOS genes in the heart. The OXPHOS structural genes and assembly factors for complexes I, II, III, IV and V stand out, as well as mitochondrial ribosomal proteins. This was not simply the product of terminal destruction of cardiac cells, since assembly genes for complex IV (COX16, COX20, COA6, SCO2 and PET100) were also regulated (Guarnieri et al., 2022). It is noteworthy that although the most striking metabolic changes at the onset of infection are observed in the lungs, the host also responds with the coordinated induction of bioenergetic gene expression in other tissues, but this process is blocked by viral action at critical transcriptional control points. While in the final stages of infection the lung is able to fully induce mitochondrial transcription, this does not occur in the heart, resulting in the generalized suppression of gene expression in cardiac mitochondria. This may explain why cardiac dysfunction is a prominent finding in COVID-19 (Nambiar et al., 2022; Xie et al., 2022). The Figure 4 summarizes the metabolic effects of SARS-CoV-2 on heart and the potential mechanisms involved. Even after the disease cure, there is increased risk of cardiovascular complications, including cardiovascular diseases, arrhythmias, and thromboembolism. Because the heart is highly dependent on mitochondrial bioenergetics, prolonged inhibition of mitochondrial bioenergetics may be an important factor that contributes to deaths associated with COVID-19.

### Modulation of hepatic metabolism in COVID-19 patients

The SARS-CoV-2 might affect the liver in two ways: direct infection from the gut to the liver (hepatic portal system) or indirect effects due to systemic inflammation, pre-existing liver disease, oxygen deprivation, and drug-related injury (Mendez-Sanchez et al., 2020; Portincasa et al., 2020; Caterino et al., 2021; Shousha et al., 2022). SARS-CoV-2 binds to the ACE-2 receptor and infects hepatocytes, which show endoplasmic reticulum expansion, mitochondrial swelling (Metawea et al., 2021), lipid metabolism alterations that include fatty degeneration, increase in lysophospholipids and fatty acids synthesis (Yan et al., 2019), and decreased glycogen granules. However, ACE-2 receptors are found in only 2.6% of the hepatocytes and in 59.7% of the cholangiocytes (Metawea et al., 2021; Shousha et al., 2022). Thus, it is more likely that the liver injury present in COVID-19 patients is caused by indirect action on hepatocytes than direct infection of the virus (Portincasa et al., 2020; Shousha et al., 2022). The main changes found in liver injury were hypoalbuminemia and an increase in serum gamma-glutamyl transferase (GGT) (Wang et al., 2020a; Kumar et al., 2020b; Metawea et al., 2021), alanine aminotransferase (ALT), and aspartate aminotransferase (AST; Kaneko et al., 2020; Kumar et al., 2020b; Wang et al., 2020d; Martinez and Franco, 2021; Metawea et al., 2021; Shousha et al., 2022), decreased CD4+ T cells and B lymphocytes (Wang and Zhang, 2020), and the relative risk of these abnormalities was higher in patients with more severe disease (Wang and Zhang, 2020; Kumar et al., 2020b).

Inflammation could be an important factor in liver damage. Cytokines, such as IL-1β, IL-6, IFN-γ-induced protein 10 (IP-10), TNF-α, IFN-γ, macrophage inflammatory protein-1α and-1β (MIP-1α and-1β), and vascular endothelial growth factor (VEGF) are raised in COVID-19 patients (Martinez and Franco, 2021). The observed high levels of IL-6 and its circulating receptor have been responsible for the deleterious effects on the liver sinusoidal endothelial cells, and blood clotting, and for contributing to liver injury in COVID-19 patients (McConnell et al., 2021). The occurrence of cytokine storm can result in hepatocellular immune-mediated damage due to viral-induced cytotoxic (CD8) T cells, and a dysregulated innate immune response (Metawea et al., 2021). In addition, this cytokine storm could be involved with changes in nitrogen metabolism, once a metabolome study in COVID-19 patients showed a positive correlation between asparagine, isoleucine, leucine, and valine with TNF-α, proline with IL-17 A and IL-17RA, threonine with IL-26, and a negative correlation of tryptophan with IL-26 (Caterino et al., 2021).

**Figure 5:**
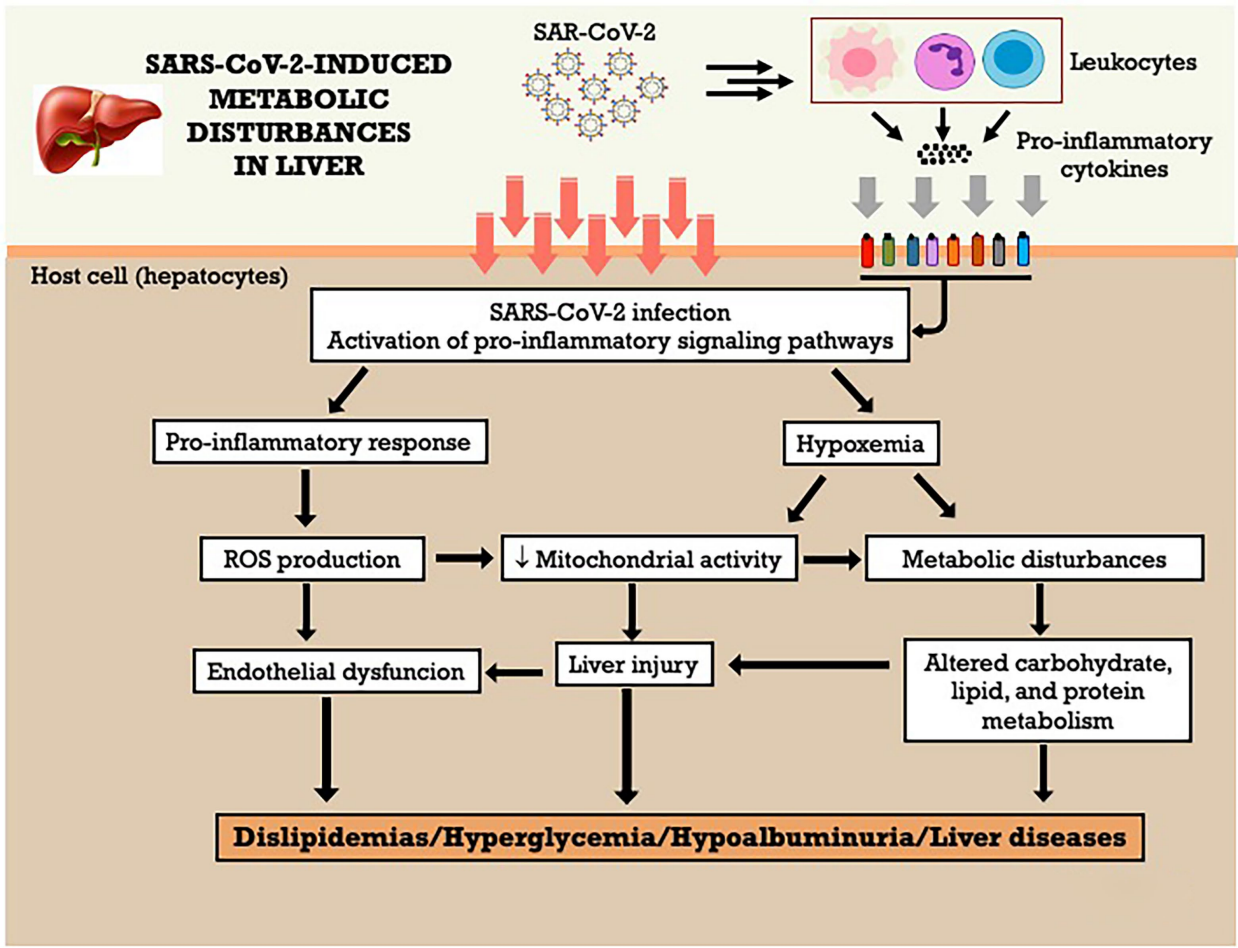
SARS-CoV-2-induced metabolic disturbances in liver. ROS, reactive oxygen species.

Patients with pre-existing liver disease may be more susceptible to liver damage from SARS-CoV-2. For example, nonalcoholic fatty liver disease (NAFLD) represents a risk factor for the greater severity of SARS-COV-2 infection (Portincasa et al., 2020; Martinez and Franco, 2021). About 2–11% of COVID-19 patients had underlying chronic liver disease, and hepatic dysfunction has been seen in 14–53% of patients with COVID-19, particularly in severe disease cases (Jothimani et al., 2020). In the other hand, ineffective gas exchange causes a hypoxic environment, and this oxygen imbalance can lead to lactate accumulation. Caterino et al. (2021) identified a pattern of increasing serum levels of lactate according to the severity of SARS-COV-2 infection in a cohort of 52 hospitalized COVID-19 patients, classified mild, moderate, and severe. The serum metabolome indicates alterations of glycolysis/gluconeogenesis, D-glutamine e D-glutamate metabolism, nitrogen metabolism, and purine and pyrimidine metabolism in the three populations of COVID-19 patients. Moreover, a positive correlation between the increase of serum lactate and the increase of succinic acid, xanthine, ornithine, and glutamate on the three levels of severity of disease suggests a connection between hypoxemia, oxidative stress, and dyslipidemia in COVID-19 patients, which can negatively affect the mitochondrial energy metabolism and detoxification process in the liver. The Figure 5 summarizes the metabolic effects of SARS-CoV-2 on liver and the potential mechanisms involved. Furthermore, according to the authors, the increased levels of ornithine, the main metabolite of the urea cycle, and the high levels of aspartate and glutamate observed in moderate and severe COVID-19 patients, which showed a positive correlation with serum lactate levels, could be signs of altered hepatic nitrogen metabolism, oxygen imbalance and tissue injury induced by SARS-COV-2 infection (Caterino et al., 2021).

### Gastrointestinal changes in COVID-19

The proper functioning of the gastrointestinal tract (GIT) helps to protect against the invasion of pathogenic microorganisms (Oliva et al., 2021). However, impairments in GIT function are associated with worsening in the progression of several diseases and infections, including COVID-19. Studies indicate an association between gastrointestinal symptoms (e.g., diarrhea, nausea, loss of appetite, gastrointestinal bleeding, abdominal discomfort) and the severity of COVID-19.

Giron et al. (2021) conducted a study with 80 patients (20 control individuals without COVID-19 and 60 patients with COVID-19, being 20 with severe, 20 with moderate, 20 with mild symptoms). They measured plasma markers of intestinal permeability, translocation of microorganisms, and inflammation and how these markers correlate with the severity of the disease. The authors observed that the disease severity is related to an increased concentration of zonulin (a tight junction protein) in the plasma of the severely ill group. The increase in intestinal permeability could facilitate the translocation of microorganisms and their metabolites, confirmed by the rise in lipopolysaccharide-binding protein (LPB) and beta-glucan, which showed high concentrations in individuals in a severe condition compared with those in a mild illness and group control. In addition, elevated inflammatory markers and immune system activation correlate with microorganism translocation and intestinal barrier impairment (Giron et al., 2021).

Oliva et al. (2021) reported increased intestinal permeability during SARS-CoV-2 infection. The present study recorded the plasma concentration of zonulin and LPS in 81 patients with COVID-19 and 81 controls with thrombotic events. Positive COVID-19 patients had high concentrations of zonulin and LPS. Among the 81 patients in the infected group, 14% had thrombotic events. The results suggested that increased intestinal permeability and endotoxemia are associated with thrombosis during the new coronavirus infection (Oliva et al., 2021).

The intestinal inflammation observed in COVID-19 patients is associated with the SARS-CoV-2 S protein. Intestinal tissue samples from positive patients who had gastrointestinal symptoms were analyzed. The inflammatory condition was highly pronounced in the small intestine and accompanied by interstitial edema, vasodilation, and hemorrhage. In patients with gastrointestinal symptoms, there was an increase in VEGF concentration. It was also observed that VEGF was associated with vasodilation and interstitial edema, disease progression even at its early stage in the COVID-19 group with gastrointestinal symptoms (Zeng et al., 2022).

One of the reasons that potentially explain the role of intestine in the progression of a disease that mainly affects the respiratory tract is the intestine-lung axis. This bidirectional communication axis allows for interaction between the two organs, and although the mechanisms remain not fully elucidated, various studies point out the participation of intestinal and pulmonary microbiota (Allali et al., 2021). In addition to the inflammatory condition and alteration of intestinal permeability, infection with the new coronavirus affects the intestinal microbiota (Kim, 2021). Studies that evaluated the composition of the microbiota of individuals positive for COVID-19 point out some changes, in particular, to a picture of dysbiosis and decreased diversity, which can extend even after the end of the infection (Yeoh et al., 2021).

COVID-19 can also contribute to the hyperglycemia development observed during the SARS-CoV-2 infection. It has been observed that ACE2 regulates the sodium-glucose-linked transporter-1 (SGLT1) activity in the intestine, which affects glucose absorption and, therefore, directly impacts glycemia (Kumar et al., 2020a). SGLT1 inhibitors are one of the drug classes used to treat diabetes mellitus through reducing glucose absorption in the small intestine (Song et al., 2016). During coronavirus infection, there is a reduction of ACE2 receptor expression in cell membrane as a result of virus binding (Imai et al., 2008). The downregulation of ACE2 receptor expression promotes SGLT1 upregulation in gastrointestinal epithelial cells, increasing intestinal glucose absorption and consequently blood glucose levels (Koufakis et al., 2021). This mechanism negatively impacts the prognosis of newly diagnosed diabetes mellitus induced by COVID-19 (Lim et al., 2021; Gorjao et al., 2022).

**Figure 6:**
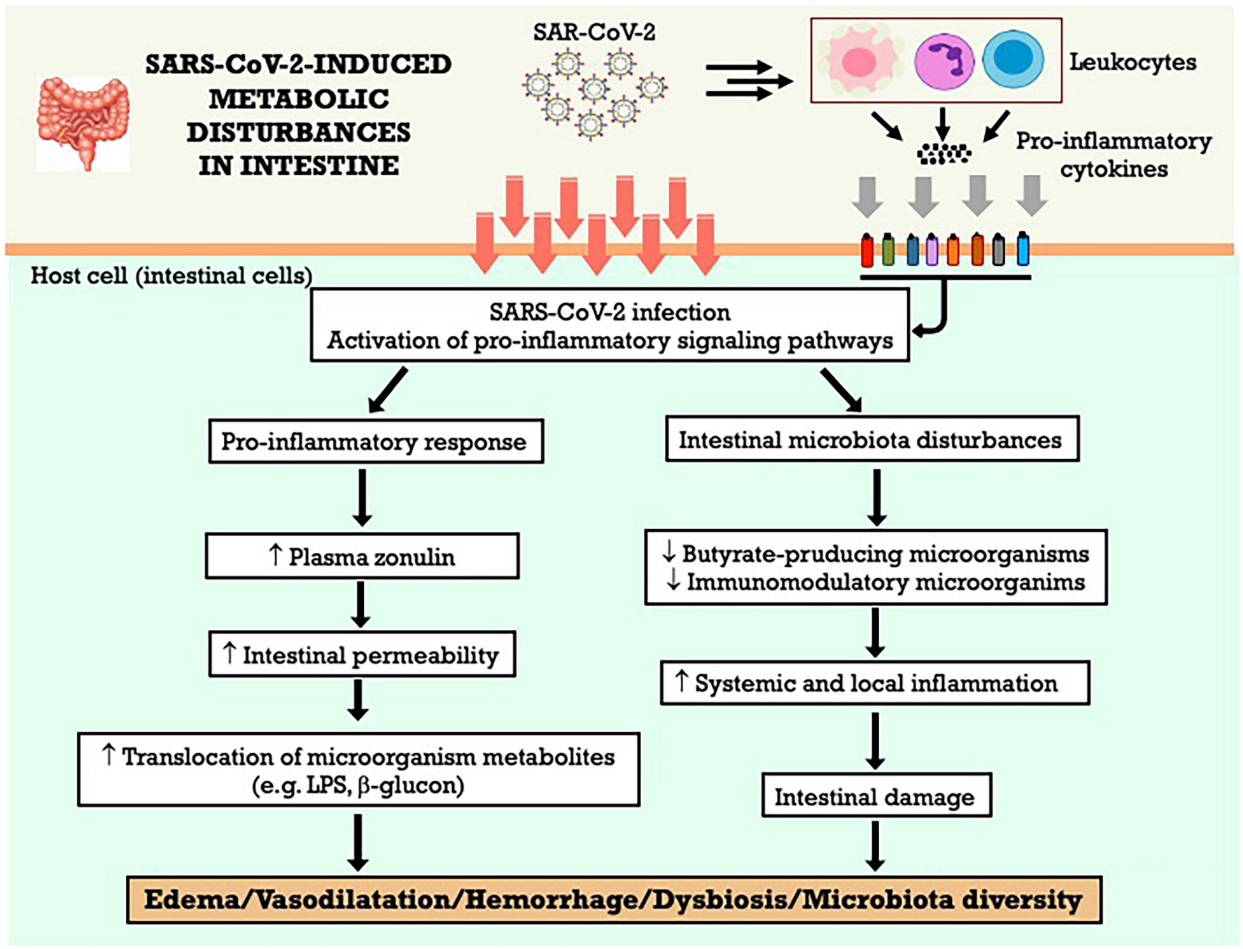
SARS-CoV-2-induced metabolic disturbances in gastrointestinal system. LPS, lipopolysaccharide.

Dysbiosis, or imbalance, of the intestinal microbiota in patients with COVID-19, was characterized by a decrease in immunomodulatory microorganisms and associated not only with an increase in plasma markers of inflammation and tissue damage but also with the severity of the disease (Yeoh et al., 2021). The results also suggest a decrease in butyrate-producing microorganisms, such as *Faecalibacterium prausnitzii* (Zuo et al., 2020; Yeoh et al., 2021). Concerning the changes reported in the gastrointestinal tract, SARS-CoV-2 infection is related to alterations in the intestinal microbiota, with a decrease in its diversity and dysbiosis, an increase in inflammation and intestinal permeability, and markers of translocation of microorganisms. These changes in the intestine may contribute to systemic inflammation, which would explain the relationship between gastrointestinal symptoms and COVID-19 severity. The Figure 6 summarizes the metabolic effects of SARS-CoV-2 on gastrointestinal system and the potential mechanisms involved.

## Concluding remarks

**Figure 7:**
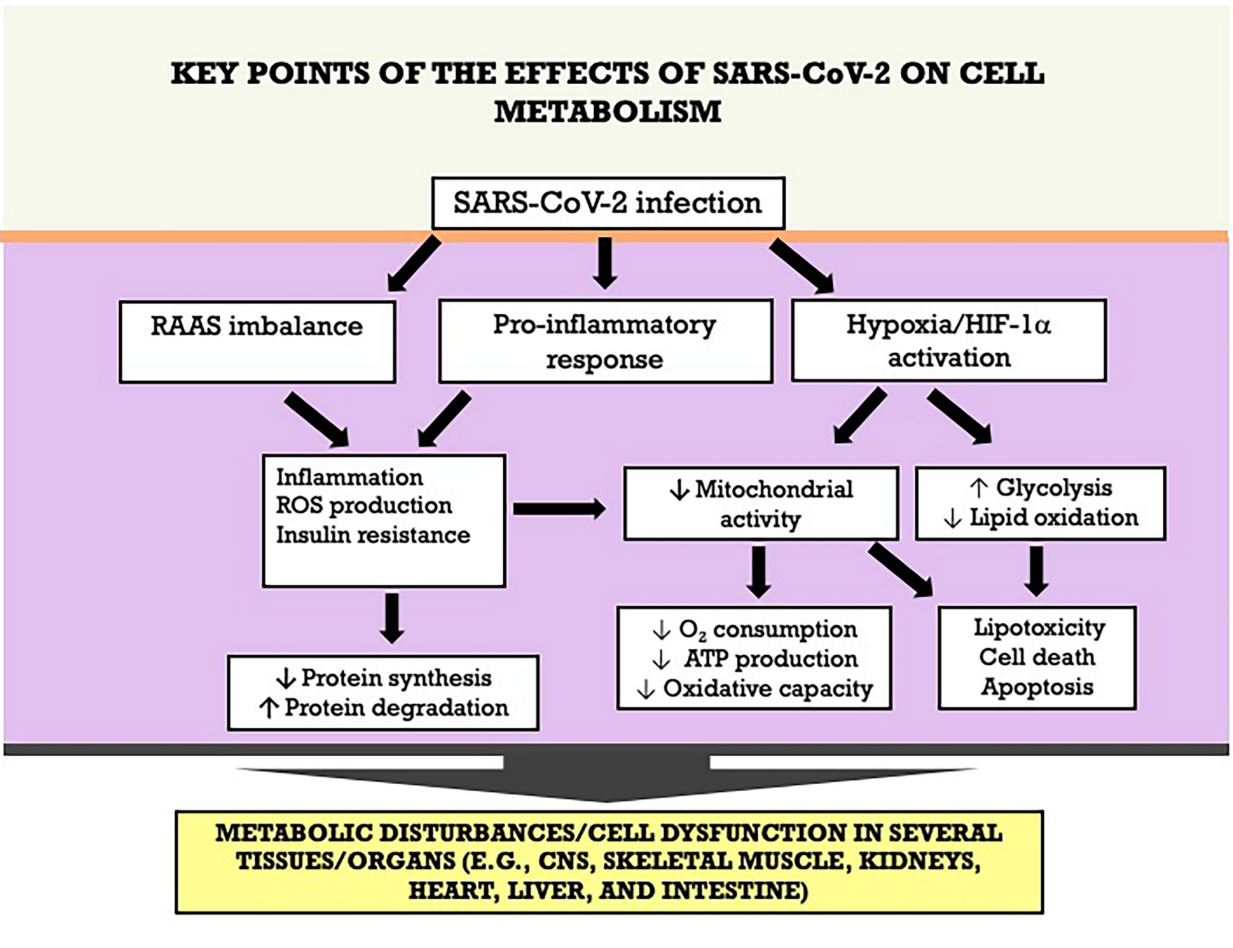
Key points of the effects of SARS-CoV-2 on cell metabolism an tissue/organ dysfunction. ATP adenosine triphosphate; HIF-1α, hypoxia-inducible factor-1 alpha; RAAS, renin-angiotensin-aldosterone system; ROS, reactive oxygen species.

COVID-19 is a multifactorial disease affecting several organs and systems, including lungs, kidneys, skeletal muscle, heart, intestine, and liver. Studies are underway to identify the main deleterious effects on metabolism in these different organs affected by SAR-CoV-2. It is known that its pathogenesis occurs through different mechanisms, including activation of inflammatory pathways, oxidative stress, and the RAAS. Metabolic disturbances are exacerbated when these mechanisms are associated, potentially resulting in tissue injury or dysfunction. The key points of the effects of SARS-CoV-2 on cell metabolism and tissue/organ dysfunction are summarized in the Figure 7. Therefore, one of the focuses for combating the disease is the understanding of the mechanisms involved in the modulation of cellular metabolism (with potential reflex in its function), aiming at the development of interventions to attenuate the deleterious metabolic effects caused by the virus.

## Author contributions

All authors attended the criteria to justify the authorship. Specifically, all authors were involved in the manuscript elaboration, literature review, and writing of the different parts of the text. All authors contributed to the article and approved the submitted version.

## Funding

The authors of this study are supported by grants from the São Paulo Research Foundation (FAPESP, São Paulo, SP, Brazil; 2018/09868–7, 2018/07283–1, and 2021/00200–6), the Coordination for the Improvement of Higher Education Personnel (CAPES, Brasilia, Brazil), the National Council for Scientific and Technological Development (CNPq, Brasilia, Brazil) and the Pro-Rectory of Post-Graduate and Research of the Cruzeiro do Sul University (PRPGP/Cruzeiro do Sul, São Paulo, SP, Brazil).

## Conflict of interest

The authors declare that the research was conducted in the absence of any commercial or financial relationships that could be construed as a potential conflict of interest.

## Publisher’s note

All claims expressed in this article are solely those of the authors and do not necessarily represent those of their affiliated organizations, or those of the publisher, the editors and the reviewers. Any product that may be evaluated in this article, or claim that may be made by its manufacturer, is not guaranteed or endorsed by the publisher.

## References

[ref1] Abdi A. Jalilian M. Sarbarzeh P. A. Vlaisavljevic Z. (2020). Diabetes and COVID-19: A systematic review on the current evidences. Diabetes Res. Clin. Pract. 166:108347. doi: 10.1016/j.diabres.2020.108347, PMID: 32711003 PMC7375314

[ref2] Abdullahi A. Candan S. A. Abba M. A. Bello A. H. Alshehri M. A. Afamefuna Victor E.. (2020). Neurological and musculoskeletal features of COVID-19: A systematic review and meta-analysis. Front. Neurol. 11:687. doi: 10.3389/fneur.2020.00687, PMID: 32676052 PMC7333777

[ref3] Aiyegbusi O. L. Hughes S. E. Turner G. Rivera S. C. Mcmullan C. Chandan J. S.. (2021). Symptoms, complications and management of long COVID: a review. J. R. Soc. Med. 114, 428–442. doi: 10.1177/01410768211032850, PMID: 34265229 PMC8450986

[ref4] Akarsu C. Karabulut M. Aydin H. Sahbaz N. A. Dural A. C. Yegul D.. (2022). Association between acute pancreatitis and COVID-19: could pancreatitis be the missing piece of the puzzle about increased mortality rates? J. Investig. Surg. 35, 119–125. doi: 10.1080/08941939.2020.1833263, PMID: 33138658

[ref5] Aksu U. Demirci C. Ince C. (2011). The pathogenesis of acute kidney injury and the toxic triangle of oxygen, reactive oxygen species and nitric oxide. Contrib. Nephrol. 174, 119–128. doi: 10.1159/000329249, PMID: 21921616

[ref6] Alberti P. Beretta S. Piatti M. Karantzoulis A. Piatti M. L. Santoro P.. (2020). Guillain-Barre syndrome related to COVID-19 infection. Neurol Neuroimmunol Neuroinflamm 7:e741. doi: 10.1212/NXI.0000000000000741, PMID: 32350026 PMC7217652

[ref7] Ali A. M. Kunugi H. (2021). Skeletal muscle damage in COVID-19: A call for action. Medicina (Kaunas), 57. 10.3390/medicina57040372 PMC8069858 33921429

[ref8] Al-Kuraishy H. M. Al-Gareeb A. I. Alblihed M. Guerreiro S. G. Cruz-Martins N. Batiha G. E. (2021). COVID-19 in relation to hyperglycemia and diabetes mellitus. Front Cardiovasc Med 8:644095. doi: 10.3389/fcvm.2021.644095, PMID: 34124187 PMC8189260

[ref9] Allali I. Bakri Y. Amzazi S. Ghazal H. (2021). Gut-Lung Axis in COVID-19. Interdiscip Perspect Infect Dis 2021:6655380. doi: 10.1155/2021/6655380 33777139 PMC7979298

[ref10] Ameres M. Brandstetter S. Toncheva A. A. Kabesch M. Leppert D. Kuhle J.. (2020). Association of neuronal injury blood marker neurofilament light chain with mild-to-moderate COVID-19. J. Neurol. 267, 3476–3478. doi: 10.1007/s00415-020-10050-y, PMID: 32647900 PMC7345451

[ref11] Andrade Silva M. Silva A D. A. Do Amaral M. A. Fragas M. G. Camara N. O. S. (2021). Metabolic alterations in SARS-CoV-2 infection and its implication in kidney dysfunction. Front. Physiol. 12:624698. doi: 10.3389/fphys.2021.624698, PMID: 33716771 PMC7947848

[ref12] Aschman T. Schneider J. Greuel S. Meinhardt J. Streit S. Goebel H. H.. (2021). Association between SARS-CoV-2 infection and immune-mediated myopathy in patients who have died. JAMA Neurol. 78, 948–960. doi: 10.1001/jamaneurol.2021.2004, PMID: 34115106 PMC12507457

[ref13] Ashraf U. M. Abokor A. A. Edwards J. M. Waigi E. W. Royfman R. S. Hasan S. A.. (2021). SARS-CoV-2, ACE2 expression, and systemic organ invasion. Physiol. Genomics 53, 51–60. doi: 10.1152/physiolgenomics.00087.2020, PMID: 33275540 PMC7900915

[ref14] Bartolini D. Stabile A. M. Bastianelli S. Giustarini D. Pierucci S. Busti C.. (2021). SARS-CoV2 infection impairs the metabolism and redox function of cellular glutathione. Redox Biol. 45:102041. doi: 10.1016/j.redox.2021.102041, PMID: 34146958 PMC8190457

[ref15] Basile D. P. Anderson M. D. Sutton T. A. (2012). Pathophysiology of acute kidney injury. Compr. Physiol. 2, 1303–1353. doi: 10.1002/cphy.c110041, PMID: 23798302 PMC3919808

[ref16] Bastin J. Cambon N. Thompson M. Lowry O. H. Burch H. B. (1987). Change in energy reserves in different segments of the nephron during brief ischemia. Kidney Int. 31, 1239–1247. doi: 10.1038/ki.1987.137, PMID: 3613402

[ref17] Baughn L. B. Sharma N. Elhaik E. Sekulic A. Bryce A. H. Fonseca R. (2020). Targeting TMPRSS2 in SARS-CoV-2 infection. Mayo Clin. Proc. 95, 1989–1999. doi: 10.1016/j.mayocp.2020.06.018, PMID: 32861340 PMC7368885

[ref18] Bekassy Z. Lopatko Fagerstrom I. Bader M. Karpman D. (2022). Crosstalk between the renin-angiotensin, complement and kallikrein-kinin systems in inflammation. Nat. Rev. Immunol. 22, 411–428. doi: 10.1038/s41577-021-00634-8, PMID: 34759348 PMC8579187

[ref19] Benhar M. (2020). Oxidants, antioxidants and thiol redox switches in the control of regulated cell death pathways. Antioxidants (Basel) 9, 1–20. doi: 10.3390/antiox9040309 PMC7222211 32290499

[ref20] Bernard-Valnet R. Pizzarotti B. Anichini A. Demars Y. Russo E. Schmidhauser M.. (2020). Two patients with acute meningoencephalitis concomitant with SARS-CoV-2 infection. Eur. J. Neurol. 27, e43–e44. doi: 10.1111/ene.14298, PMID: 32383343 PMC7267660

[ref21] Berni A. Malandrino D. Parenti G. Maggi M. Poggesi L. Peri A. (2020). Hyponatremia, IL-6, and SARS-CoV-2 (COVID-19) infection: may all fit together? J. Endocrinol. Investig. 43, 1137–1139. doi: 10.1007/s40618-020-01301-w, PMID: 32451971 PMC7246958

[ref22] Berti V. Mosconi L. Pupi A. (2014). Brain: normal variations and benign findings in fluorodeoxyglucose-PET/computed tomography imaging. PET Clin 9, 129–140. doi: 10.1016/j.cpet.2013.10.006, PMID: 24772054 PMC3998066

[ref23] Bijuklic K. Jennings P. Kountchev J. Hasslacher J. Aydin S. Sturn D.. (2007). Migration of leukocytes across an endothelium-epithelium bilayer as a model of renal interstitial inflammation. Am. J. Physiol. Cell Physiol. 293, C486–C492. doi: 10.1152/ajpcell.00419.2006, PMID: 17428840

[ref24] Bobulescu I. A. Dubree M. Zhang J. Mcleroy P. Moe O. W. (2008). Effect of renal lipid accumulation on proximal tubule Na+/H+ exchange and ammonium secretion. Am. J. Physiol. Renal Physiol. 294, F1315–F1322. doi: 10.1152/ajprenal.00550.2007, PMID: 18417539 PMC2861570

[ref25] Bode B. Garrett V. Messler J. Mcfarland R. Crowe J. Booth R.. (2020). Glycemic characteristics and clinical outcomes of COVID-19 patients hospitalized in the United States. J. Diabetes Sci. Technol. 14, 813–821. doi: 10.1177/1932296820924469, PMID: 32389027 PMC7673150

[ref26] Bojkova D. Klann K. Koch B. Widera M. Krause D. Ciesek S.. (2020). Proteomics of SARS-CoV-2-infected host cells reveals therapy targets. Nature 583, 469–472. doi: 10.1038/s41586-020-2332-7, PMID: 32408336 PMC7616921

[ref27] Bonventre J. V. Zuk A. (2004). Ischemic acute renal failure: an inflammatory disease? Kidney Int. 66, 480–485. doi: 10.1111/j.1523-1755.2004.761_2.x 15253693

[ref28] Brann D. H. Tsukahara T. Weinreb C. Lipovsek M. Van Den Berge K. Gong B.. (2020). Non-neuronal expression of SARS-CoV-2 entry genes in the olfactory system suggests mechanisms underlying COVID-19-associated anosmia. Sci. Adv. 6:801. doi: 10.1126/sciadv.abc5801, PMID: 32937591 PMC10715684

[ref29] Braun F. Lutgehetmann M. Pfefferle S. Wong M. N. Carsten A. Lindenmeyer M. T.. (2020). SARS-CoV-2 renal tropism associates with acute kidney injury. Lancet 396, 597–598. doi: 10.1016/S0140-6736(20)31759-1, PMID: 32818439 PMC7431179

[ref30] Bruzzone C. Bizkarguenaga M. Gil-Redondo R. Diercks T. Arana E. Garcia De Vicuna A.. (2020). SARS-CoV-2 infection dysregulates the Metabolomic and Lipidomic profiles of serum. iScience 23:101645. doi: 10.1016/j.isci.2020.101645, PMID: 33043283 PMC7534591

[ref31] Butowt R. Bilinska K. (2020). SARS-CoV-2: olfaction, brain infection, and the urgent need for clinical samples allowing earlier virus detection. ACS Chem. Neurosci. 11, 1200–1203. doi: 10.1021/acschemneuro.0c00172, PMID: 32283006

[ref32] Cabello-Verrugio C. Morales M. G. Rivera J. C. Cabrera D. Simon F. (2015). Renin-angiotensin system: an old player with novel functions in skeletal muscle. Med. Res. Rev. 35, 437–463. doi: 10.1002/med.21343, PMID: 25764065

[ref33] Cai Y. Shi S. Yang F. Yi B. Chen X. Li J.. (2020). Fasting blood glucose level is a predictor of mortality in patients with COVID-19 independent of diabetes history. Diabetes Res. Clin. Pract. 169:108437. doi: 10.1016/j.diabres.2020.108437, PMID: 32920103 PMC7482587

[ref34] Camini F. C. Da Silva Caetano C. C. Almeida L. T. De Brito Magalhaes C. L. (2017). Implications of oxidative stress on viral pathogenesis. Arch. Virol. 162, 907–917. doi: 10.1007/s00705-016-3187-y 28039563

[ref35] Cantuti-Castelvetri L. Ojha R. Pedro L. D. Djannatian M. Franz J. Kuivanen S.. (2020). Neuropilin-1 facilitates SARS-CoV-2 cell entry and infectivity. Science 370, 856–860. doi: 10.1126/science.abd2985, PMID: 33082293 PMC7857391

[ref36] Casqueiro J. Casqueiro J. Alves C. (2012). Infections in patients with diabetes mellitus: A review of pathogenesis. Indian J Endocrinol Metab 16, S27–S36. doi: 10.4103/2230-8210.94253 22701840 PMC3354930

[ref37] Caterino M. Costanzo M. Fedele R. Cevenini A. Gelzo M. Minno, A D. I.. (2021). The serum metabolome of moderate and severe COVID-19 patients reflects possible liver alterations involving carbon and nitrogen metabolism. Int. J. Mol. Sci. 22:548. doi: 10.3390/ijms22179548, PMID: 34502454 PMC8431319

[ref38] Ceriello A. (2020). Hyperglycemia and COVID-19: what was known and what is really new? Diabetes Res. Clin. Pract. 167:108383. doi: 10.1016/j.diabres.2020.108383, PMID: 32853690 PMC7445137

[ref39] Chachlaki K. Prevot V. (2020). Nitric oxide signalling in the brain and its control of bodily functions. Br. J. Pharmacol. 177, 5437–5458. doi: 10.1111/bph.14800, PMID: 31347144 PMC7707094

[ref40] Channappanavar R. Perlman S. (2017). Pathogenic human coronavirus infections: causes and consequences of cytokine storm and immunopathology. Semin. Immunopathol. 39, 529–539. doi: 10.1007/s00281-017-0629-x, PMID: 28466096 PMC7079893

[ref41] Chavez-Reyes J. Escarcega-Gonzalez C. E. Chavira-Suarez E. Leon-Buitimea A. Vazquez-Leon P. Morones-Ramirez J. R.. (2021). Susceptibility for some infectious diseases in patients with diabetes: the key role of Glycemia. Front. Public Health 9:559595. doi: 10.3389/fpubh.2021.559595, PMID: 33665182 PMC7921169

[ref42] Chen L. Li X. Chen M. Feng Y. Xiong C. (2020). The ACE2 expression in human heart indicates new potential mechanism of heart injury among patients infected with SARS-CoV-2. Cardiovasc. Res. 116, 1097–1100. doi: 10.1093/cvr/cvaa078, PMID: 32227090 PMC7184507

[ref43] Cheng Y. Luo R. Wang K. Zhang M. Wang Z. Dong L.. (2020). Kidney disease is associated with in-hospital death of patients with COVID-19. Kidney Int. 97, 829–838. doi: 10.1016/j.kint.2020.03.005, PMID: 32247631 PMC7110296

[ref44] Chernyak B. V. Popova E. N. Prikhodko A. S. Grebenchikov O. A. Zinovkina L. A. Zinovkin R. A. (2020). COVID-19 and oxidative stress. Biochemistry (Mosc) 85, 1543–1553. doi: 10.1134/S0006297920120068, PMID: 33705292 PMC7768996

[ref45] Chew M. S. Blixt P. J. Ahman R. Engerstrom L. Andersson H. Berggren R. K.. (2021). National outcomes and characteristics of patients admitted to Swedish intensive care units for COVID-19: A registry-based cohort study. Eur. J. Anaesthesiol. 38, 335–343. doi: 10.1097/EJA.0000000000001459, PMID: 33534266

[ref46] Chiloiro S. Capoluongo E. D. Tartaglione T. Giampietro A. Bianchi A. Giustina A.. (2019). The changing clinical Spectrum of Hypophysitis. Trends Endocrinol. Metab. 30, 590–602. doi: 10.1016/j.tem.2019.06.004, PMID: 31345630

[ref47] Choi K. D. Kim J. S. Park S. H. Kim Y. K. Kim S. E. Smitt P. S. (2006). Cerebellar hypermetabolism in paraneoplastic cerebellar degeneration. J. Neurol. Neurosurg. Psychiatry 77, 525–528. doi: 10.1136/jnnp.2005.075325, PMID: 16543536 PMC2077501

[ref48] Chougar L. Shor N. Weiss N. Galanaud D. Leclercq D. Mathon B.. (2020). Retrospective observational study of brain MRI findings in patients with acute SARS-CoV-2 infection and neurologic manifestations. Radiology 297, E313–E323. doi: 10.1148/radiol.2020202422, PMID: 32677875 PMC7370354

[ref49] Codo A. C. Davanzo G. G. Monteiro L. B. Souza D. E. F G. Muraro S. P.. (2020). Elevated glucose levels favor SARS-CoV-2 infection and monocyte response through a HIF-1alpha/glycolysis-dependent Axis. Cell Metab. 32:e5. doi: 10.1016/j.cmet.2020.07.007 PMC7462530 32877692

[ref50] Connors J. M. Levy J. H. (2020). COVID-19 and its implications for thrombosis and anticoagulation. Blood 135, 2033–2040. doi: 10.1182/blood.2020006000, PMID: 32339221 PMC7273827

[ref51] Cummings M. J. Baldwin M. R. Abrams D. Jacobson S. D. Meyer B. J. Balough E. M.. (2020). Epidemiology, clinical course, and outcomes of critically ill adults with COVID-19 in new York City: a prospective cohort study. Lancet 395, 1763–1770. doi: 10.1016/S0140-6736(20)31189-2, PMID: 32442528 PMC7237188

[ref52] Daly J. L. Simonetti B. Klein K. Chen K. E. Williamson M. K. Anton-Plagaro C.. (2020). Neuropilin-1 is a host factor for SARS-CoV-2 infection. Science 370, 861–865. doi: 10.1126/science.abd3072, PMID: 33082294 PMC7612957

[ref53] de Rivero Vaccari J. C. Dietrich W. D. Keane R. W. de Rivero Vaccari J. P. (2020). The Inflammasome in times of COVID-19. Front. Immunol. 11:583373. doi: 10.3389/fimmu.2020.583373 33149733 PMC7580384

[ref54] Delafontaine P. Yoshida T. (2016). The renin-angiotensin system and the biology of skeletal muscle: mechanisms of muscle wasting in chronic disease states. Trans. Am. Clin. Climatol. Assoc. 127, 245–258. PMID: 28066057 PMC5216488

[ref55] Delorme C. Paccoud O. Kas A. Hesters A. Bombois S. Shambrook P.. (2020). COVID-19-related encephalopathy: a case series with brain FDG-positron-emission tomography/computed tomography findings. Eur. J. Neurol. 27, 2651–2657. doi: 10.1111/ene.14478, PMID: 32881133 PMC7461074

[ref56] Dillard L. R. Wase N. Ramakrishnan G. Park J. J. Sherman N. E. Carpenter R.. (2022). Leveraging metabolic modeling to identify functional metabolic alterations associated with COVID-19 disease severity. Metabolomics 18:51. doi: 10.1007/s11306-022-01904-9, PMID: 35819731 PMC9273921

[ref57] Disser N. P. De Micheli A. J. Schonk M. M. Konnaris M. A. Piacentini A. N. Edon D. L.. (2020). Musculoskeletal consequences of COVID-19. J. Bone Joint Surg. Am. 102, 1197–1204. doi: 10.2106/JBJS.20.00847, PMID: 32675661 PMC7508274

[ref58] Dungan K. M. Braithwaite S. S. Preiser J. C. (2009). Stress hyperglycaemia. Lancet 373, 1798–1807. doi: 10.1016/S0140-6736(09)60553-5, PMID: 19465235 PMC3144755

[ref59] Eckardt K. U. Bernhardt W. M. Weidemann A. Warnecke C. Rosenberger C. Wiesener M. S.. (2005). Role of hypoxia in the pathogenesis of renal disease. Kidney Int. Suppl. 68, S46–S51. doi: 10.1111/j.1523-1755.2005.09909.x 16336576

[ref60] Espindola O. M. Brandao C. O. Gomes Y. C. P. Siqueira M. Soares C. N. Lima M.. (2021). Cerebrospinal fluid findings in neurological diseases associated with COVID-19 and insights into mechanisms of disease development. Int. J. Infect. Dis. 102, 155–162. doi: 10.1016/j.ijid.2020.10.044, PMID: 33127503 PMC7591319

[ref61] Faubel S. Edelstein C. L. (2016). Mechanisms and mediators of lung injury after acute kidney injury. Nat. Rev. Nephrol. 12, 48–60. doi: 10.1038/nrneph.2015.158, PMID: 26434402

[ref62] Fenouillet E. Barbouche R. Jones I. M. (2007). Cell entry by enveloped viruses: redox considerations for HIV and SARS-coronavirus. Antioxid. Redox Signal. 9, 1009–1034. doi: 10.1089/ars.2007.1639, PMID: 17567241

[ref63] Fernandes I. G. De Brito C. A. Dos Reis V. M. S. Sato M. N. Pereira N. Z. (2020). SARS-CoV-2 and other respiratory viruses: what does oxidative stress have to do with it? Oxidative Med. Cell. Longev. 2020:8844280. doi: 10.1155/2020/8844280 PMC7757116 33381273

[ref64] Ferrandi P. J. Alway S. E. Mohamed J. S. (2020). The interaction between SARS-CoV-2 and ACE2 may have consequences for skeletal muscle viral susceptibility and myopathies. J. Appl. Physiol. 1985, 864–867. doi: 10.1152/japplphysiol.00321.2020 PMC7832004 32673162

[ref65] Frantz E. D. C. Prodel E. Braz I. D. Giori I. G. Bargut T. C. L. Magliano D. C.. (2018). Modulation of the renin-angiotensin system in white adipose tissue and skeletal muscle: focus on exercise training. Clin. Sci. (Lond.) 132, 1487–1507. doi: 10.1042/CS20180276, PMID: 30037837

[ref66] Fujii S. Akaike T. (2013). Redox signaling by 8-nitro-cyclic guanosine monophosphate: nitric oxide-and reactive oxygen species-derived electrophilic messenger. Antioxid. Redox Signal. 19, 1236–1246. doi: 10.1089/ars.2012.5067, PMID: 23157314

[ref67] Fukuda R. Zhang H. Kim J. W. Shimoda L. Dang C. V. Semenza G. L. (2007). HIF-1 regulates cytochrome oxidase subunits to optimize efficiency of respiration in hypoxic cells. Cells 129, 111–122. doi: 10.1016/j.cell.2007.01.047, PMID: 17418790

[ref68] Gangadharan C. Ahluwalia R. Sigamani A. (2021). Diabetes and COVID-19: role of insulin resistance as a risk factor for COVID-19 severity. World J. Diabetes 12, 1550–1562. doi: 10.4239/wjd.v12.i9.1550, PMID: 34630907 PMC8472493

[ref69] Gao Y. L. Du Y. Zhang C. Cheng C. Yang H. Y. Jin Y. F.. (2020). Role of renin-angiotensin system in acute lung injury caused by viral infection. Infect Drug Resist 13, 3715–3725. doi: 10.2147/IDR.S265718, PMID: 33116692 PMC7585866

[ref70] Garg R. K. Paliwal V. K. Gupta A. (2021). Encephalopathy in patients with COVID-19: A review. J. Med. Virol. 93, 206–222. doi: 10.1002/jmv.26207 32558956

[ref71] Giron L. B. Dweep H. Yin X. Wang H. Damra M. Goldman A. R.. (2021). Corrigendum: plasma markers of disrupted gut permeability in severe COVID-19 patients. Front. Immunol. 12:779064. doi: 10.3389/fimmu.2021.779064, PMID: 34671365 PMC8522493

[ref72] Gomez-Mesa J. E. Galindo-Coral S. Montes M. C. Munoz Martin A. J. (2021). Thrombosis and coagulopathy in COVID-19. Curr. Probl. Cardiol. 46:100742. doi: 10.1016/j.cpcardiol.2020.100742, PMID: 33243440 PMC7605852

[ref73] Gonzalez A. Orozco-Aguilar J. Achiardi O. Simon F. Cabello-Verrugio C. (2020). SARS-CoV-2/renin-angiotensin system: deciphering the clues for a couple with potentially harmful effects on skeletal muscle. Int. J. Mol. Sci. 21:904. doi: 10.3390/ijms21217904, PMID: 33114359 PMC7663203

[ref74] Gonzalez-Flecha B. Boveris A. (1995). Mitochondrial sites of hydrogen peroxide production in reperfused rat kidney cortex. Biochim. Biophys. Acta 1243, 361–366. doi: 10.1016/0304-4165(94)00160-Y, PMID: 7727510

[ref75] Gorjao R. Hirabara S. M. Masi L. N. Serdan T. D. A. Gritte R. B. Hatanaka E.. (2022). Poor prognosis indicators of type-2 diabetic COVID-19 patients. Braz. J. Med. Biol. Res. 55:e11819. doi: 10.1590/1414-431x2022e11819 35766706 PMC9224823

[ref76] Groop L. C. Bonadonna R. C. Delprato S. Ratheiser K. Zyck K. Ferrannini E.. (1989). Glucose and free fatty acid metabolism in non-insulin-dependent diabetes mellitus. Evidence for multiple sites of insulin resistance. J. Clin. Invest. 84, 205–213. doi: 10.1172/JCI114142, PMID: 2661589 PMC303971

[ref77] Guan W. J. Ni Z. Y. Hu Y. Liang W. H. Ou C. Q. He J. X.. (2020). Clinical characteristics of coronavirus disease 2019 in China. N. Engl. J. Med. 382, 1708–1720. doi: 10.1056/NEJMoa2002032, PMID: 32109013 PMC7092819

[ref78] Guarnieri J. W. Dybas J. M. Fazelinia H. Kim M. S. Frere J. Zhang Y.. (2022). Targeted down regulation of Core mitochondrial genes during SARS-CoV-2 infection. bioRxiv. doi: 10.1101/2022.02.19.481089

[ref79] Guedj E. Million M. Dudouet P. Tissot-Dupont H. Bregeon F. Cammilleri S.. (2021). (18)F-FDG brain PET hypometabolism in post-SARS-CoV-2 infection: substrate for persistent/delayed disorders? Eur. J. Nucl. Med. Mol. Imaging 48, 592–595. doi: 10.1007/s00259-020-04973-x, PMID: 32728799 PMC7391029

[ref80] Haggstrom L. R. Nelson J. A. Wegner E. A. Caplan G. A. (2017). 2-(18)F-fluoro-2-deoxyglucose positron emission tomography in delirium. J. Cereb. Blood Flow Metab. 37, 3556–3567. doi: 10.1177/0271678X17701764, PMID: 28350285 PMC5669345

[ref81] Han Q. Zheng B. Daines L. Sheikh A. (2022). Long-term sequelae of COVID-19: A systematic review and meta-analysis of one-year follow-up studies on post-COVID symptoms. Pathogens 11:269. doi: 10.3390/pathogens11020269, PMID: 35215212 PMC8875269

[ref82] Harapan B. N. Yoo H. J. (2021). Neurological symptoms, manifestations, and complications associated with severe acute respiratory syndrome coronavirus 2 (SARS-CoV-2) and coronavirus disease 19 (COVID-19). J. Neurol. 268, 3059–3071. doi: 10.1007/s00415-021-10406-y, PMID: 33486564 PMC7826147

[ref83] Hassanein M. Radhakrishnan Y. Sedor J. Vachharajani T. Vachharajani V. T. Augustine J.. (2020). COVID-19 and the kidney. Cleve. Clin. J. Med. 87, 619–631. doi: 10.3949/ccjm.87a.20072 33004323

[ref84] Heaton N. S. Randall G. (2010). Dengue virus-induced autophagy regulates lipid metabolism. Cell Host Microbe 8, 422–432. doi: 10.1016/j.chom.2010.10.006, PMID: 21075353 PMC3026642

[ref85] Hejbol E. K. Harbo T. Agergaard J. Madsen L. B. Pedersen T. H. Ostergaard L. J.. (2022). Myopathy as a cause of fatigue in long-term post-COVID-19 symptoms: evidence of skeletal muscle histopathology. Eur. J. Neurol. 29, 2832–2841. doi: 10.1111/ene.15435, PMID: 35661354 PMC9348124

[ref86] Helms J. Kremer S. Merdji H. Clere-JEHL R. Schenck M. Kummerlen C.. (2020). Neurologic features in severe SARS-CoV-2 infection. N. Engl. J. Med. 382, 2268–2270. doi: 10.1056/NEJMc2008597, PMID: 32294339 PMC7179967

[ref87] Hersh E. V. Wolff M. Moore P. A. Theken K. N. Daniell H. (2022). A pair of "ACEs". J. Dent. Res. 101, 5–10. doi: 10.1177/00220345211047510, PMID: 34689655 PMC8721725

[ref88] Hill P. Shukla D. Tran M. G. Aragones J. Cook H. T. Carmeliet P.. (2008). Inhibition of hypoxia inducible factor hydroxylases protects against renal ischemia-reperfusion injury. J. Am. Soc. Nephrol. 19, 39–46. doi: 10.1681/ASN.2006090998, PMID: 18178798 PMC2391027

[ref89] Hirsch J. S. Ng J. H. Ross D. W. Sharma P. Shah H. H. Barnett R. L.. (2020). Acute kidney injury in patients hospitalized with COVID-19. Kidney Int. 98, 209–218. doi: 10.1016/j.kint.2020.05.006, PMID: 32416116 PMC7229463

[ref90] Hoke T. S. Douglas I. S. Klein C. L. He Z. Fang W. Thurman J. M.. (2007). Acute renal failure after bilateral nephrectomy is associated with cytokine-mediated pulmonary injury. J. Am. Soc. Nephrol. 18, 155–164. doi: 10.1681/ASN.2006050494, PMID: 17167117

[ref91] Hollstein T. Schulte D. M. Schulz J. Gluck A. Ziegler A. G. Bonifacio E.. (2020). Autoantibody-negative insulin-dependent diabetes mellitus after SARS-CoV-2 infection: a case report. Nat. Metab. 2, 1021–1024. doi: 10.1038/s42255-020-00281-8, PMID: 32879473

[ref92] Hosp J. A. Dressing A. Blazhenets G. Bormann T. Rau A. Schwabenland M.. (2021). Cognitive impairment and altered cerebral glucose metabolism in the subacute stage of COVID-19. Brain 144, 1263–1276. doi: 10.1093/brain/awab009, PMID: 33822001 PMC8083602

[ref93] Hu B. Huang S. Yin L. (2021). The cytokine storm and COVID-19. J. Med. Virol. 93, 250–256. doi: 10.1002/jmv.26232, PMID: 32592501 PMC7361342

[ref94] Huang C. Wang Y. Li X. Ren L. Zhao J. Hu Y.. (2020). Clinical features of patients infected with 2019 novel coronavirus in Wuhan, China. Lancet 395, 497–506. doi: 10.1016/S0140-6736(20)30183-5, PMID: 31986264 PMC7159299

[ref95] Iadecola C. Anrather J. Kamel H. (2020). Effects of COVID-19 on the nervous system. Cells 183:e1. doi: 10.1016/j.cell.2020.08.028 PMC7437501 32882182

[ref96] Imai Y. Kuba K. Penninger J. M. (2008). The discovery of angiotensin-converting enzyme 2 and its role in acute lung injury in mice. Exp. Physiol. 93, 543–548. doi: 10.1113/expphysiol.2007.040048, PMID: 18448662 PMC7197898

[ref97] Jin Y. Ji W. Yang H. Chen S. Zhang W. Duan G. (2020). Endothelial activation and dysfunction in COVID-19: from basic mechanisms to potential therapeutic approaches. Signal Transduct. Target. Ther. 5:293. doi: 10.1038/s41392-020-00454-7, PMID: 33361764 PMC7758411

[ref98] Jin M. Tong Q. (2020). Rhabdomyolysis as potential late complication associated with COVID-19. Emerg. Infect. Dis. 26, 1618–1620. doi: 10.3201/eid2607.200445, PMID: 32197060 PMC7323559

[ref99] Jo E. K. Kim J. K. Shin D. M. Sasakawa C. (2016). Molecular mechanisms regulating NLRP3 inflammasome activation. Cell. Mol. Immunol. 13, 148–159. doi: 10.1038/cmi.2015.95, PMID: 26549800 PMC4786634

[ref100] Jones D. P. Sies H. (2015). The redox code. Antioxid. Redox Signal. 23, 734–746. doi: 10.1089/ars.2015.6247, PMID: 25891126 PMC4580308

[ref101] Jothimani D. Venugopal R. Abedin M. F. Kaliamoorthy I. Rela M. (2020). COVID-19 and the liver. J. Hepatol. 73, 1231–1240. doi: 10.1016/j.jhep.2020.06.006, PMID: 32553666 PMC7295524

[ref102] Kai H. Kai M. (2020). Interactions of coronaviruses with ACE2, angiotensin II, and RAS inhibitors-lessons from available evidence and insights into COVID-19. Hypertens. Res. 43, 648–654. doi: 10.1038/s41440-020-0455-8, PMID: 32341442 PMC7184165

[ref103] Kanberg N. Ashton N. J. Andersson L. M. Yilmaz A. Lindh M. Nilsson S.. (2020). Neurochemical evidence of astrocytic and neuronal injury commonly found in COVID-19. Neurology 95, e1754–e1759. doi: 10.1212/WNL.0000000000010111, PMID: 32546655

[ref104] Kaneko S. Kurosaki M. Nagata K. Taki R. Ueda K. Hanada S.. (2020). Liver injury with COVID-19 based on gastrointestinal symptoms and pneumonia severity. PLoS One 15:e0241663. doi: 10.1371/journal.pone.0241663, PMID: 33147270 PMC7641400

[ref105] Kanova M. Kohout P. (2022). Molecular mechanisms underlying intensive care unit-acquired weakness and sarcopenia. Int. J. Mol. Sci. 23:396. doi: 10.3390/ijms23158396, PMID: 35955530 PMC9368893

[ref106] Kas A. Soret M. Pyatigorskaya N. Habert M. O. Hesters A. Le Guennec L.. (2022). Correction to: the cerebral network of COVID-19-related encephalopathy: a longitudinal voxel-based 18F-FDG-PET study. Eur. J. Nucl. Med. Mol. Imaging 49:3304. doi: 10.1007/s00259-022-05812-x, PMID: 35570216 PMC9108017

[ref107] Kautzky-Willer A. Kaleta M. Lindner S. D. Leutner M. Thurner S. Klimek P. (2022). Sex differences in clinical characteristics and outcomes of patients with SARS-CoV-2-infection admitted to intensive care units in Austria. J Pers Med 12:517. doi: 10.3390/jpm12040517, PMID: 35455633 PMC9026885

[ref108] Kazakou P. Paschou S. A. Psaltopoulou T. Gavriatopoulou M. Korompoki E. Stefanaki K.. (2021). Early and late endocrine complications of COVID-19. Endocr. Connect. 10, R229–R239. doi: 10.1530/EC-21-0184, PMID: 34424853 PMC8494407

[ref109] Khovidhunkit W. Kim M. S. Memon R. A. Shigenaga J. K. Moser A. H. Feingold K. R.. (2004). Effects of infection and inflammation on lipid and lipoprotein metabolism: mechanisms and consequences to the host. J. Lipid Res. 45, 1169–1196. doi: 10.1194/jlr.R300019-JLR200, PMID: 15102878

[ref110] Kim H. S. (2021). Do an altered gut microbiota and an associated leaky gut affect COVID-19 severity? mBio 12, 1–9. doi: 10.1128/mBio.03022-20 PMC7845625 33436436

[ref111] Kim N. Y. Ha E. Moon J. S. Lee Y. H. Choi E. Y. (2020). Response: acute hyperglycemic crises with coronavirus Disease-19: case reports. Diabetes Metab. J. 44, 484–485. doi: 10.4093/dmj.2020.0129, PMID: 32347027 PMC7188962

[ref112] Kim S. M. Kim Y. G. Jeong K. H. Lee S. H. Lee T. W. Ihm C. G.. (2012). Angiotensin II-induced mitochondrial Nox4 is a major endogenous source of oxidative stress in kidney tubular cells. PLoS One 7:e39739. doi: 10.1371/journal.pone.0039739, PMID: 22808054 PMC3392275

[ref113] Koufakis T. Metallidis S. Zebekakis P. Kotsa K. (2021). Intestinal Sglt1 as a therapeutic target in COVID-19-related diabetes: A "two-edged sword" hypothesis. Br. J. Clin. Pharmacol. 87, 3643–3646. doi: 10.1111/bcp.14800, PMID: 33684969 PMC8251113

[ref114] Kozlov A. V. Lancaster J. R. Meszaros A. T. Weidinger A. (2017). Mitochondria-meditated pathways of organ failure upon inflammation. Redox Biol. 13, 170–181. doi: 10.1016/j.redox.2017.05.017, PMID: 28578275 PMC5458092

[ref115] Kumar A. Faiq M. A. Pareek V. Raza K. Narayan R. K. Prasoon P.. (2020a). Relevance of SARS-CoV-2 related factors ACE2 and TMPRSS2 expressions in gastrointestinal tissue with pathogenesis of digestive symptoms, diabetes-associated mortality, and disease recurrence in COVID-19 patients. Med. Hypotheses 144:110271. doi: 10.1016/j.mehy.2020.110271, PMID: 33254575 PMC7487155

[ref116] Kumar M. P. Mishra S. Jha D. K. Shukla J. Choudhury A. Mohindra R.. (2020b). Coronavirus disease (COVID-19) and the liver: a comprehensive systematic review and meta-analysis. Hepatol. Int. 14, 711–722. doi: 10.1007/s12072-020-10071-9, PMID: 32623633 PMC7335221

[ref117] Lavillette D. Barbouche R. Yao Y. Boson B. Cosset F. L. Jones I. M.. (2006). Significant redox insensitivity of the functions of the SARS-CoV spike glycoprotein: comparison with HIV envelope. J. Biol. Chem. 281, 9200–9204. doi: 10.1074/jbc.M512529200, PMID: 16418166 PMC7982606

[ref118] Lazarus G. Audrey J. Wangsaputra V. K. Tamara A. Tahapary D. L. (2021). High admission blood glucose independently predicts poor prognosis in COVID-19 patients: A systematic review and dose-response meta-analysis. Diabetes Res. Clin. Pract. 171:108561. doi: 10.1016/j.diabres.2020.108561, PMID: 33310127 PMC7725108

[ref119] Lee L. Y. W. Cazier J. B. Starkey T. Briggs S. E. W. Arnold R. Bisht V.. (2020). COVID-19 prevalence and mortality in patients with cancer and the effect of primary tumour subtype and patient demographics: a prospective cohort study. Lancet Oncol. 21, 1309–1316. doi: 10.1016/S1470-2045(20)30442-3, PMID: 32853557 PMC7444972

[ref120] Lee M. H. Perl D. P. Nair G. Li W. Maric D. Murray H.. (2021). Microvascular injury in the brains of patients with Covid-19. N. Engl. J. Med. 384, 481–483. doi: 10.1056/NEJMc2033369, PMID: 33378608 PMC7787217

[ref121] Letko M. Marzi A. Munster V. (2020). Functional assessment of cell entry and receptor usage for SARS-CoV-2 and other lineage B betacoronaviruses. Nat. Microbiol. 5, 562–569. doi: 10.1038/s41564-020-0688-y, PMID: 32094589 PMC7095430

[ref122] Li F. (2015). Receptor recognition mechanisms of coronaviruses: a decade of structural studies. J. Virol. 89, 1954–1964. doi: 10.1128/JVI.02615-14, PMID: 25428871 PMC4338876

[ref123] Li X. Fang P. Mai J. Choi E. T. Wang H. Yang X. F. (2013). Targeting mitochondrial reactive oxygen species as novel therapy for inflammatory diseases and cancers. J. Hematol. Oncol. 6:19. doi: 10.1186/1756-8722-6-19, PMID: 23442817 PMC3599349

[ref124] Li S. Jiang L. Li X. Lin F. Wang Y. Li B.. (2020b). Clinical and pathological investigation of patients with severe COVID-19. JCI Insight 5:070. doi: 10.1172/jci.insight.138070, PMID: 32427582 PMC7406259

[ref125] Li M. Y. Li L. Zhang Y. Wang X. S. (2020a). Expression of the SARS-CoV-2 cell receptor gene ACE2 in a wide variety of human tissues. Infect. Dis. Poverty 9:45. doi: 10.1186/s40249-020-00662-x, PMID: 32345362 PMC7186534

[ref126] Li Z. Xu X. Leng X. He M. Wang J. Cheng S.. (2017). Roles of reactive oxygen species in cell signaling pathways and immune responses to viral infections. Arch. Virol. 162, 603–610. doi: 10.1007/s00705-016-3130-2, PMID: 27848013

[ref127] Lim S. Bae J. H. Kwon H. S. Nauck M. A. (2021). COVID-19 and diabetes mellitus: from pathophysiology to clinical management. Nat. Rev. Endocrinol. 17, 11–30. doi: 10.1038/s41574-020-00435-4, PMID: 33188364 PMC7664589

[ref128] Liu B. Li M. Zhou Z. Guan X. Xiang Y. (2020a). Can we use interleukin-6 (IL-6) blockade for coronavirus disease 2019 (COVID-19)-induced cytokine release syndrome (CRS)? J. Autoimmun. 111:102452. doi: 10.1016/j.jaut.2020.102452, PMID: 32291137 PMC7151347

[ref129] Liu F. Liu F. Wang L. (2021). COVID-19 and cardiovascular diseases. J. Mol. Cell Biol. 13, 161–167. doi: 10.1093/jmcb/mjaa064, PMID: 33226078 PMC7717280

[ref130] Liu F. Long X. Zhang B. Zhang W. Chen X. Zhang Z. (2020b). ACE2 expression in pancreas may cause pancreatic damage after SARS-CoV-2 infection. Clin. Gastroenterol. Hepatol. 18:e2. doi: 10.1016/j.cgh.2020.04.040 32334082 PMC7194639

[ref131] Lucas C. Wong P. Klein J. Castro T. B. R. Silva J. Sundaram M.. (2020). Longitudinal analyses reveal immunological misfiring in severe COVID-19. Nature 584, 463–469. doi: 10.1038/s41586-020-2588-y, PMID: 32717743 PMC7477538

[ref132] Madanineishaboori A. Moshrefiaraghi D. Mohamed Ali K. Toloui A. Yousefifard M. Hosseini M. (2020). Central nervous system complications in COVID-19 patients; a systematic review and meta-analysis based on current evidence. Arch Acad Emerg Med 8:e62. doi: 10.22037/aaem.v8i1.798 33134959 PMC7587989

[ref133] Mao L. Jin H. Wang M. Hu Y. Chen S. He Q.. (2020). Neurologic manifestations of hospitalized patients with coronavirus disease 2019 in Wuhan, China. JAMA Neurol. 77, 683–690. doi: 10.1001/jamaneurol.2020.1127, PMID: 32275288 PMC7149362

[ref134] Marietta M. Ageno W. Artoni A. De Candia E. Gresele P. Marchetti M.. (2020). COVID-19 and haemostasis: a position paper from Italian society on thrombosis and Haemostasis (SISET). Blood Transfus. 18, 167–169. doi: 10.2450/2020.0083-20, PMID: 32281926 PMC7250682

[ref135] Martinez M. A. Franco S. (2021). Impact of COVID-19 in liver disease progression. Hepatol Commun 5, 1138–1150. doi: 10.1002/hep4.1745, PMID: 34533001 PMC8239862

[ref136] Matschke J. Lutgehetmann M. Hagel C. Sperhake J. P. Schroder A. S. Edler C.. (2020). Neuropathology of patients with COVID-19 in Germany: a post-mortem case series. Lancet Neurol. 19, 919–929. doi: 10.1016/S1474-4422(20)30308-2, PMID: 33031735 PMC7535629

[ref137] Mcconnell M. J. Kawaguchi N. Kondo R. Sonzogni A. Licini L. Valle C.. (2021). Liver injury in COVID-19 and IL-6 trans-signaling-induced endotheliopathy. J. Hepatol. 75, 647–658. doi: 10.1016/j.jhep.2021.04.050, PMID: 33991637 PMC8285256

[ref138] Mendez-Sanchez N. Valencia-Rodriguez A. Qi X. Yoshida E. M. Romero-Gomez M. George J.. (2020). What has the COVID-19 pandemic taught us so far? Addressing the problem from a Hepatologist's perspective. J. Clin. Transl. Hepatol. 8:0024. doi: 10.14218/JCTH.2020.00024 32309152 PMC7163687

[ref139] Metawea M. I. Yousif W. I. Moheb I. (2021). COVID 19 and liver: An A-Z literature review. Dig. Liver Dis. 53, 146–152. doi: 10.1016/j.dld.2020.09.010, PMID: 32988758 PMC7494329

[ref140] Moldogazieva N. T. Mokhosoev I. M. Feldman N. B. Lutsenko S. V. (2018). ROS and RNS signalling: adaptive redox switches through oxidative/nitrosative protein modifications. Free Radic. Res. 52, 507–543. doi: 10.1080/10715762.2018.1457217, PMID: 29589770

[ref141] Montes-Ibarra M. Oliveira C. L. P. Orsso C. E. Landi F. Marzetti E. Prado C. M. (2022). The impact of Long COVID-19 on muscle health. Clin. Geriatr. Med. 38, 545–557. doi: 10.1016/j.cger.2022.03.004, PMID: 35868672 PMC8934728

[ref142] Moreno-Altamirano M. M. B. Kolstoe S. E. Sanchez-Garcia F. J. (2019). Virus control of cell metabolism for replication and evasion of host immune responses. Front. Cell. Infect. Microbiol. 9:95. doi: 10.3389/fcimb.2019.00095, PMID: 31058096 PMC6482253

[ref143] Morris G. Bortolasci C. C. Puri B. K. Olive L. Marx W. O'neil A.. (2020). The pathophysiology of SARS-CoV-2: A suggested model and therapeutic approach. Life Sci. 258:118166. doi: 10.1016/j.lfs.2020.118166, PMID: 32739471 PMC7392886

[ref144] Mulchandani R. Lyngdoh T. Kakkar A. K. (2021). Deciphering the COVID-19 cytokine storm: systematic review and meta-analysis. Eur. J. Clin. Investig. 51:e13429. doi: 10.1111/eci.13429 33058143 PMC7646004

[ref145] Muller J. A. Gross R. Conzelmann C. Kruger J. Merle U. Steinhart J.. (2021). SARS-CoV-2 infects and replicates in cells of the human endocrine and exocrine pancreas. Nat. Metab. 3, 149–165. doi: 10.1038/s42255-021-00347-1, PMID: 33536639

[ref146] Naik E. Dixit V. M. (2011). Mitochondrial reactive oxygen species drive proinflammatory cytokine production. J. Exp. Med. 208, 417–420. doi: 10.1084/jem.20110367, PMID: 21357740 PMC3058577

[ref147] Nakahira K. Haspel J. A. Rathinam V. A. Lee S. J. Dolinay T. Lam H. C.. (2011). Autophagy proteins regulate innate immune responses by inhibiting the release of mitochondrial DNA mediated by the NALP3 inflammasome. Nat. Immunol. 12, 222–230. doi: 10.1038/ni.1980, PMID: 21151103 PMC3079381

[ref148] Nakhleh A. Shehadeh N. (2020). Interactions between antihyperglycemic drugs and the renin-angiotensin system: putative roles in COVID-19. A mini-review. Diabetes Metab Syndr 14, 509–512. doi: 10.1016/j.dsx.2020.04.040, PMID: 32388330 PMC7198998

[ref149] Nambiar L. Volodarskiy A. Tak K. A. Agoglia H. K. Zhang D. Mitlak H.. (2022). Acute COVID-19-associated decrements in left and right ventricular function predict all-cause mortality. J. Am. Soc. Echocardiogr. 35, 229–234. doi: 10.1016/j.echo.2021.10.002, PMID: 34627973 PMC8496907

[ref150] Nasiri M. J. Haddadi S. Tahvildari A. Farsi Y. Arbabi M. Hasanzadeh S.. (2020). COVID-19 clinical characteristics, and sex-specific risk of mortality: systematic review and meta-analysis. Front Med (Lausanne) 7:459. doi: 10.3389/fmed.2020.00459, PMID: 32793620 PMC7385184

[ref151] Nishida M. Kumagai Y. Ihara H. Fujii S. Motohashi H. Akaike T. (2016). Redox signaling regulated by electrophiles and reactive sulfur species. J. Clin. Biochem. Nutr. 58, 91–98. doi: 10.3164/jcbn.15-111, PMID: 27013774 PMC4788399

[ref152] Nouri-Vaskeh M. Sharifi A. Khalili N. Zand R. Sharifi A. (2020). Dyspneic and non-dyspneic (silent) hypoxemia in COVID-19: possible neurological mechanism. Clin. Neurol. Neurosurg. 198:106217. doi: 10.1016/j.clineuro.2020.106217, PMID: 32947193 PMC7480672

[ref153] Oliva A. Cammisotto V. Cangemi R. Ferro D. Miele M. C. Angelis, M D. E.. (2021). Low-grade Endotoxemia and thrombosis in COVID-19. Clin. Transl. Gastroenterol. 12:e00348. doi: 10.14309/ctg.0000000000000348, PMID: 34092777 PMC8183715

[ref154] Oz M. Lorke D. E. (2021). Multifunctional angiotensin converting enzyme 2, the SARS-CoV-2 entry receptor, and critical appraisal of its role in acute lung injury. Biomed. Pharmacother. 136:111193. doi: 10.1016/j.biopha.2020.111193, PMID: 33461019 PMC7836742

[ref155] Pal R. Bhansali A. (2020). COVID-19, diabetes mellitus and ACE2: the conundrum. Diabetes Res. Clin. Pract. 162:108132. doi: 10.1016/j.diabres.2020.108132, PMID: 32234504 PMC7118535

[ref156] Parsons N. Outsikas A. Parish A. Clohesy R. D'aprano F. Toomey F.. (2021). Modelling the anatomic distribution of neurologic events in patients with COVID-19: A systematic review of MRI findings. AJNR Am. J. Neuroradiol. 42, 1190–1195. doi: 10.3174/ajnr.A7113, PMID: 33888458 PMC8324279

[ref157] Pasini E. Corsetti G. Romano C. Scarabelli T. M. Chen-Scarabelli C. Saravolatz L.. (2021). Serum metabolic profile in patients with Long-Covid (PASC) syndrome: clinical implications. Front Med (Lausanne) 8:714426. doi: 10.3389/fmed.2021.714426, PMID: 34368201 PMC8339407

[ref158] Perez-Torres I. Soto M. E. Guarner-Lans V. Manzano-Pech L. Soria-Castro E. (2022). The possible role of Glucose-6-phosphate dehydrogenase in the SARS-CoV-2 infection. Cells 11:982. doi: 10.3390/cells11131982, PMID: 35805067 PMC9265820

[ref159] Pfoh E. R. Wozniak A. W. Colantuoni E. Dinglas V. D. Mendez-Tellez P. A. Shanholtz C.. (2016). Physical declines occurring after hospital discharge in ARDS survivors: a 5-year longitudinal study. Intensive Care Med. 42, 1557–1566. doi: 10.1007/s00134-016-4530-1, PMID: 27637716

[ref160] Pilotto A. Odolini S. Masciocchi S. Comelli A. Volonghi I. Gazzina S.. (2020). Steroid-responsive encephalitis in coronavirus disease 2019. Ann. Neurol. 88, 423–427. doi: 10.1002/ana.25783, PMID: 32418288 PMC7276848

[ref161] Portincasa P. Krawczyk M. Smyk W. Lammert F. Di Ciaula A. (2020). COVID-19 and non-alcoholic fatty liver disease: two intersecting pandemics. Eur. J. Clin. Investig. 50:e13338. doi: 10.1111/eci.13338 32589264 PMC7361203

[ref162] Porto B. N. Stein R. T. (2016). Neutrophil extracellular traps in pulmonary diseases: too much of a good thing? Front. Immunol. 7:311. doi: 10.3389/fimmu.2016.00311 27574522 PMC4983612

[ref163] Powers S. K. Morton A. B. Hyatt H. Hinkley M. J. (2018). The renin-angiotensin system and skeletal muscle. Exerc. Sport Sci. Rev. 46, 205–214. doi: 10.1249/JES.0000000000000158, PMID: 30001274 PMC6673677

[ref164] Radzinski M. Oppenheim T. Metanis N. Reichmann D. (2021). The Cys sense: thiol redox switches mediate life cycles of cellular proteins. Biomol. Ther. 11:469. doi: 10.3390/biom11030469 PMC8004198 33809923

[ref165] Rahmani-Kukia N. Abbasi A. (2021). Physiological and immunological causes of the susceptibility of chronic inflammatory patients to COVID-19 infection: focus on diabetes. Front Endocrinol (Lausanne) 12:576412. doi: 10.3389/fendo.2021.576412, PMID: 33746897 PMC7971178

[ref166] Rao S. Benzouak T. Gunpat S. Burns R. J. Tahir T. A. Jolles S.. (2022). Fatigue symptoms associated with COVID-19 in convalescent or recovered COVID-19 patients; a systematic review and meta-analysis. Ann. Behav. Med. 56, 219–234. doi: 10.1093/abm/kaab081, PMID: 34665858 PMC8574547

[ref167] Ratchford S. M. Stickford J. L. Province V. M. Stute N. Augenreich M. A. Koontz L. K.. (2021). Vascular alterations among young adults with SARS-CoV-2. Am. J. Physiol. Heart Circ. Physiol. 320, H404–H410. doi: 10.1152/ajpheart.00897.2020, PMID: 33306450 PMC8083172

[ref168] Ritz L. Segobin S. Lannuzel C. Laniepce A. Boudehent C. Cabe N.. (2019). Cerebellar Hypermetabolism in alcohol use disorder: compensatory mechanism or maladaptive plasticity? Alcohol. Clin. Exp. Res. 43, 2212–2221. doi: 10.1111/acer.14158 31373706

[ref169] Robson B. (2020). Bioinformatics studies on a function of the SARS-CoV-2 spike glycoprotein as the binding of host sialic acid glycans. Comput. Biol. Med. 122:103849. doi: 10.1016/j.compbiomed.2020.103849, PMID: 32658736 PMC7278709

[ref170] Rocheteau P. Chatre L. Briand D. Mebarki M. Jouvion G. Bardon J.. (2015). Sepsis induces long-term metabolic and mitochondrial muscle stem cell dysfunction amenable by mesenchymal stem cell therapy. Nat. Commun. 6:10145. doi: 10.1038/ncomms10145, PMID: 26666572 PMC4682118

[ref171] Rubino F. Amiel S. A. Zimmet P. Alberti G. Bornstein S. Eckel R. H.. (2020). New-onset diabetes in Covid-19. N. Engl. J. Med. 383, 789–790. doi: 10.1056/NEJMc2018688, PMID: 32530585 PMC7304415

[ref172] Ruidera E. Irazu C. E. Rajagopalan P. R. Orak J. K. Fitts C. T. Singh I. (1988). Fatty acid metabolism in renal ischemia. Lipids 23, 882–884. doi: 10.1007/BF02536209 3185124

[ref173] Saleh J. Peyssonnaux C. Singh K. K. Edeas M. (2020). Mitochondria and microbiota dysfunction in COVID-19 pathogenesis. Mitochondrion 54, 1–7. doi: 10.1016/j.mito.2020.06.008, PMID: 32574708 PMC7837003

[ref174] Sartore G. Bassani D. Ragazzi E. Traldi P. Lapolla A. Moro S. (2021). In silico evaluation of the interaction between ACE2 and SARS-CoV-2 spike protein in a hyperglycemic environment. Sci. Rep. 11:22860. doi: 10.1038/s41598-021-02297-w, PMID: 34819560 PMC8613179

[ref175] Semenza G. L. Wang G. L. (1992). A nuclear factor induced by hypoxia via de novo protein synthesis binds to the human erythropoietin gene enhancer at a site required for transcriptional activation. Mol. Cell. Biol. 12, 5447–5454. PMID: 1448077 10.1128/mcb.12.12.5447-5454.1992 PMC360482

[ref176] Shah B. R. Hux J. E. (2003). Quantifying the risk of infectious diseases for people with diabetes. Diabetes Care 26, 510–513. doi: 10.2337/diacare.26.2.510, PMID: 12547890

[ref177] Shao Y. Saredy J. Xu K. Sun Y. Saaoud F. Drummer C. T.. (2021). Endothelial immunity trained by coronavirus infections, DAMP stimulations and regulated by anti-oxidant NRF2 may contribute to inflammations, Myelopoiesis, COVID-19 cytokine storms and thromboembolism. Front. Immunol. 12:653110. doi: 10.3389/fimmu.2021.653110, PMID: 34248940 PMC8269631

[ref178] Sharma P. Uppal N. N. Wanchoo R. Shah H. H. Yang Y. Parikh R.. (2020). COVID-19-associated kidney injury: A case series of kidney biopsy findings. J. Am. Soc. Nephrol. 31, 1948–1958. doi: 10.1681/ASN.2020050699, PMID: 32660970 PMC7461689

[ref179] Shauly-Aharonov M. Shafrir A. Paltiel O. Calderon-Margalit R. Safadi R. Bicher R.. (2021). Both high and low pre-infection glucose levels associated with increased risk for severe COVID-19: new insights from a population-based study. PLoS One 16:e0254847. doi: 10.1371/journal.pone.0254847, PMID: 34293038 PMC8297851

[ref180] Shepherd S. Batra A. Lerner D. P. (2017). Review of critical illness myopathy and neuropathy. Neurohospitalist 7, 41–48. doi: 10.1177/1941874416663279, PMID: 28042370 PMC5167093

[ref181] Shete A. (2020). Urgent need for evaluating agonists of angiotensin-(1-7)/mas receptor axis for treating patients with COVID-19. Int. J. Infect. Dis. 96, 348–351. doi: 10.1016/j.ijid.2020.05.002, PMID: 32389847 PMC7204665

[ref182] Shi S. Qin M. Shen B. Cai Y. Liu T. Yang F.. (2020). Association of Cardiac Injury with Mortality in hospitalized patients with COVID-19 in Wuhan, China. JAMA Cardiol. 5, 802–810. doi: 10.1001/jamacardio.2020.0950, PMID: 32211816 PMC7097841

[ref183] Shi Y. Zeida A. Edwards C. E. Mallory M. L. Sastre S. Machado M. R.. (2022). Thiol-based chemical probes exhibit antiviral activity against SARS-CoV-2 via allosteric disulfide disruption in the spike glycoprotein. Proc. Natl. Acad. Sci. U. S. A. 119, 1–9. doi: 10.1073/pnas.2120419119 PMC8833197 35074895

[ref184] Shousha H. I. Ramadan A. Lithy R. El-Kassas M. (2022). Patterns of liver profile disturbance in patients with COVID-19. World J. Clin. Cases 10, 2063–2071. doi: 10.12998/wjcc.v10.i7.2063, PMID: 35321162 PMC8895188

[ref185] Shu S. Wang Y. Zheng M. Liu Z. Cai J. Tang C.. (2019). Hypoxia and hypoxia-inducible factors in kidney injury and repair. Cells 8:207. doi: 10.3390/cells8030207, PMID: 30823476 PMC6468851

[ref186] Sies H. (2017). Hydrogen peroxide as a central redox signaling molecule in physiological oxidative stress: oxidative eustress. Redox Biol. 11, 613–619. doi: 10.1016/j.redox.2016.12.035, PMID: 28110218 PMC5256672

[ref187] Singbartl K. Formeck C. L. Kellum J. A. (2019). Kidney-Immune System Crosstalk in AKI. Semin. Nephrol. 39, 96–106. doi: 10.1016/j.semnephrol.2018.10.007, PMID: 30606411

[ref188] Singhal T. (2020). A review of coronavirus Disease-2019 (COVID-19). The Indian Journal of Pediatrics 87, 281–286. doi: 10.1007/s12098-020-03263-6, PMID: 32166607 PMC7090728

[ref189] Smith L. E. (2020). High-density lipoproteins and acute kidney injury. Semin. Nephrol. 40, 232–242. doi: 10.1016/j.semnephrol.2020.01.013, PMID: 32303285

[ref190] Soares M. N. Eggelbusch M. Naddaf E. Gerrits K. H. L. Van Der Schaaf M. Van Den Borst B.. (2022). Skeletal muscle alterations in patients with acute Covid-19 and post-acute sequelae of Covid-19. J. Cachexia. Sarcopenia Muscle 13, 11–22. doi: 10.1002/jcsm.12896, PMID: 34997689 PMC8818659

[ref191] Soler M. J. Wysocki J. Batlle D. (2013). ACE2 alterations in kidney disease. Nephrol. Dial. Transplant. 28, 2687–2697. doi: 10.1093/ndt/gft320, PMID: 23956234 PMC3811059

[ref192] Song E. Bartley C. M. Chow R. D. Ngo T. T. Jiang R. Zamecnik C. R.. (2021). Divergent and self-reactive immune responses in the CNS of COVID-19 patients with neurological symptoms. Cell Rep Med 2:100288. doi: 10.1016/j.xcrm.2021.100288, PMID: 33969321 PMC8091032

[ref193] Song P. Onishi A. Koepsell H. Vallon V. (2016). Sodium glucose cotransporter SGLT1 as a therapeutic target in diabetes mellitus. Expert Opin. Ther. Targets 20, 1109–1125. doi: 10.1517/14728222.2016.1168808, PMID: 26998950 PMC5045806

[ref194] Stasi A. Franzin R. Fiorentino M. Squiccimarro E. Castellano G. Gesualdo L. (2021). Multifaced roles of HDL in sepsis and SARS-CoV-2 infection: renal implications. Int. J. Mol. Sci. 22:980. doi: 10.3390/ijms22115980, PMID: 34205975 PMC8197836

[ref195] Su H. Lei C. T. Zhang C. (2017). Interleukin-6 signaling pathway and its role in kidney disease: An update. Front. Immunol. 8:405. doi: 10.3389/fimmu.2017.00405, PMID: 28484449 PMC5399081

[ref196] Su H. Yang M. Wan C. Yi L. X. Tang F. Zhu H. Y.. (2020). Renal histopathological analysis of 26 postmortem findings of patients with COVID-19 in China. Kidney Int. 98, 219–227. doi: 10.1016/j.kint.2020.04.003, PMID: 32327202 PMC7194105

[ref197] Suh J. Mukerji S. S. Collens S. I. Padera R. F. Jr. Pinkus G. S. Amato A. A.. (2021). Skeletal muscle and peripheral nerve histopathology in COVID-19. Neurology 97, e849–e858. doi: 10.1212/WNL.0000000000012344, PMID: 34099523

[ref198] Suhail S. Zajac J. Fossum C. Lowater H. Mccracken C. Severson N.. (2020). Role of oxidative stress on SARS-CoV (SARS) and SARS-CoV-2 (COVID-19) infection: A review. Protein J. 39, 644–656. doi: 10.1007/s10930-020-09935-8, PMID: 33106987 PMC7587547

[ref199] Sykes D. L. Holdsworth L. Jawad N. Gunasekera P. Morice A. H. Crooks M. G. (2021). Post-COVID-19 symptom burden: what is Long-COVID and how should we manage it? Lung 199, 113–119. doi: 10.1007/s00408-021-00423-z, PMID: 33569660 PMC7875681

[ref200] Tajbakhsh A. Gheibi Hayat S. M. Taghizadeh H. Akbari A. Inabadi M. Savardashtaki A.. (2021). COVID-19 and cardiac injury: clinical manifestations, biomarkers, mechanisms, diagnosis, treatment, and follow up. Expert Rev. Anti-Infect. Ther. 19, 345–357. doi: 10.1080/14787210.2020.1822737, PMID: 32921216

[ref201] Tang N. Bai H. Chen X. Gong J. Li D. Sun Z. (2020). Anticoagulant treatment is associated with decreased mortality in severe coronavirus disease 2019 patients with coagulopathy. J. Thromb. Haemost. 18, 1094–1099. doi: 10.1111/jth.14817, PMID: 32220112 PMC9906401

[ref202] Tang X. Uhl S. Zhang T. Xue D. Li B. Vandana J. J.. (2021). SARS-CoV-2 infection induces beta cell transdifferentiation. Cell Metab. 33:e7. doi: 10.1016/j.cmet.2021.05.015 PMC8133495 34081913

[ref203] Tay M. Z. Poh C. M. Renia L. Macary P. A. Ng L. F. P. (2020). The trinity of COVID-19: immunity, inflammation and intervention. Nat. Rev. Immunol. 20, 363–374. doi: 10.1038/s41577-020-0311-8, PMID: 32346093 PMC7187672

[ref204] Thachil J. Tang N. Gando S. Falanga A. Cattaneo M. Levi M.. (2020). ISTH interim guidance on recognition and management of coagulopathy in COVID-19. J. Thromb. Haemost. 18, 1023–1026. doi: 10.1111/jth.14810, PMID: 32338827 PMC9906133

[ref205] Thai M. Graham N. A. Braas D. Nehil M. Komisopoulou E. Kurdistani S. K.. (2014). Adenovirus E4ORF1-induced MYC activation promotes host cell anabolic glucose metabolism and virus replication. Cell Metab. 19, 694–701. doi: 10.1016/j.cmet.2014.03.009, PMID: 24703700 PMC4294542

[ref206] Thaker S. K. Ch'ng J. Christofk H. R. (2019). Viral hijacking of cellular metabolism. BMC Biol. 17:59. doi: 10.1186/s12915-019-0678-9, PMID: 31319842 PMC6637495

[ref207] Twaddell S. H. Baines K. J. Grainge C. Gibson P. G. (2019). The emerging role of neutrophil extracellular traps in respiratory disease. Chest 156, 774–782. doi: 10.1016/j.chest.2019.06.012 31265835

[ref208] Uchida S. Endou H. (1988). Substrate specificity to maintain cellular ATP along the mouse nephron. Am. J. Phys. 255, F977–F983. 10.1152/ajprenal.1988.255.5.F977 2847554

[ref209] Vaduganathan M. Vardeny O. Michel T. Mcmurray J. J. V. Pfeffer M. A. Solomon S. D. (2020). Renin-angiotensin-aldosterone system inhibitors in patients with Covid-19. N. Engl. J. Med. 382, 1653–1659. doi: 10.1056/NEJMsr2005760, PMID: 32227760 PMC7121452

[ref210] Vanhorebeek I. Latronico N. Van Den Berghe G. (2020). ICU-acquired weakness. Intensive Care Med. 46, 637–653. doi: 10.1007/s00134-020-05944-4, PMID: 32076765 PMC7224132

[ref211] Vastag L. Koyuncu E. Grady S. L. Shenk T. E. Rabinowitz J. D. (2011). Divergent effects of human cytomegalovirus and herpes simplex virus-1 on cellular metabolism. PLoS Pathog. 7:e1002124. doi: 10.1371/journal.ppat.1002124, PMID: 21779165 PMC3136460

[ref212] Venkatachalam M. A. Griffin K. A. Lan R. Geng H. Saikumar P. Bidani A. K. (2010). Acute kidney injury: a springboard for progression in chronic kidney disease. Am. J. Physiol. Renal Physiol. 298, F1078–F1094. doi: 10.1152/ajprenal.00017.2010, PMID: 20200097 PMC2867413

[ref213] Viana F. D. Cardoso B. I. D. S. Aragão V. M. S. Thomaz Branco C. H. L. G. Silva R. V. A. D. Simplicio F. G. (2022). Proposta de ligante Para o receptor de Neuropilina-1, alvo molecular do SARS-CoV-2 / proposed ligand for the Neuropilin-1 receptor, molecular target of SARS-CoV-2. Brazilian Journal of Development 8, 46091–46108. doi: 10.34117/bjdv8n6-228

[ref214] Wang L. Foer D. Macphaul E. Lo Y. C. Bates D. W. Zhou L. (2022). PASCLex: A comprehensive post-acute sequelae of COVID-19 (PASC) symptom lexicon derived from electronic health record clinical notes. J. Biomed. Inform. 125:103951. doi: 10.1016/j.jbi.2021.103951, PMID: 34785382 PMC8590503

[ref215] Wang J. Hajizadeh N. Moore E. E. Mcintyre R. C. Moore P. K. Veress L. A.. (2020b). Tissue plasminogen activator (tPA) treatment for COVID-19 associated acute respiratory distress syndrome (ARDS): A case series. J. Thromb. Haemost. 18, 1752–1755. doi: 10.1111/jth.14828, PMID: 32267998 PMC7262152

[ref216] Wang D. Hu B. Hu C. Zhu F. Liu X. Zhang J.. (2020a). Clinical characteristics of 138 hospitalized patients with 2019 novel coronavirus-infected pneumonia in Wuhan, China. JAMA 323, 1061–1069. doi: 10.1001/jama.2020.1585, PMID: 32031570 PMC7042881

[ref217] Wang Y. Liu S. Liu H. Li W. Lin F. Jiang L.. (2020d). SARS-CoV-2 infection of the liver directly contributes to hepatic impairment in patients with COVID-19. J. Hepatol. 73, 807–816. doi: 10.1016/j.jhep.2020.05.002, PMID: 32437830 PMC7211738

[ref218] Wang S. Ma P. Zhang S. Song S. Wang Z. Ma Y.. (2020c). Fasting blood glucose at admission is an independent predictor for 28-day mortality in patients with COVID-19 without previous diagnosis of diabetes: a multi-Centre retrospective study. Diabetologia 63, 2102–2111. doi: 10.1007/s00125-020-05209-1, PMID: 32647915 PMC7347402

[ref219] Wang Z. Zhang W. (2020). The crosstalk between hypoxia-inducible factor-1alpha and microRNAs in acute kidney injury. Exp. Biol. Med. (Maywood) 245, 427–436. doi: 10.1177/1535370220902696, PMID: 31996035 PMC7082886

[ref220] Wen H. Gwathmey J. K. Xie L. H. (2012). Oxidative stress-mediated effects of angiotensin II in the cardiovascular system. World J Hypertens 2, 34–44. doi: 10.5494/wjh.v2.i4.34, PMID: 24587981 PMC3936474

[ref221] West A. P. Khoury-Hanold W. Staron M. Tal M. C. Pineda C. M. Lang S. M.. (2015). Mitochondrial Dna stress primes the antiviral innate immune response. Nature 520, 553–557. doi: 10.1038/nature14156, PMID: 25642965 PMC4409480

[ref222] Wu J. Huang J. Zhu G. Wang Q. Lv Q. Huang Y.. (2020b). Elevation of blood glucose level predicts worse outcomes in hospitalized patients with COVID-19: a retrospective cohort study. BMJ Open Diabetes Res. Care 8:e001476. doi: 10.1136/bmjdrc-2020-001476, PMID: 32503812 PMC7298690

[ref223] Wu C. T. Lidsky P. V. Xiao Y. Lee I. T. Cheng R. Nakayama T.. (2021). SARS-CoV-2 infects human pancreatic beta cells and elicits beta cell impairment. Cell Metab. 33:e5. doi: 10.1016/j.cmet.2021.05.013 PMC8130512 34081912

[ref224] Wu F. Zhao S. Yu B. Chen Y. M. Wang W. Song Z. G.. (2020a). A new coronavirus associated with human respiratory disease in China. Nature 579, 265–269. doi: 10.1038/s41586-020-2008-3, PMID: 32015508 PMC7094943

[ref225] Xie Y. Xu E. Bowe B. Al-Aly Z. (2022). Long-term cardiovascular outcomes of COVID-19. Nat. Med. 28, 583–590. doi: 10.1038/s41591-022-01689-3, PMID: 35132265 PMC8938267

[ref226] Yan B. Chu H. Yang D. Sze K. H. Lai P. M. Yuan S.. (2019). Characterization of the Lipidomic profile of human coronavirus-infected cells: implications for lipid metabolism remodeling upon coronavirus replication. Viruses 11:073. doi: 10.3390/v11010073, PMID: 30654597 PMC6357182

[ref227] Yang J. Chen T. Zhou Y. (2021a). Mediators of SARS-CoV-2 entry are preferentially enriched in cardiomyocytes. Hereditas 158:4. doi: 10.1186/s41065-020-00168-4, PMID: 33397514 PMC7781406

[ref228] Yang L. Han Y. Nilsson-Payant B. E. Gupta V. Wang P. Duan X.. (2020). A human pluripotent stem cell-based platform to study SARS-CoV-2 tropism and model virus infection in human cells and organoids. Cell Stem Cell 27:e7, 125–136. doi: 10.1016/j.stem.2020.06.015 PMC7303620 32579880

[ref229] Yang P. Wang N. Wang J. Luo A. Gao F. Tu Y. (2021b). Admission fasting plasma glucose is an independent risk factor for 28-day mortality in patients with COVID-19. J. Med. Virol. 93, 2168–2176. doi: 10.1002/jmv.26608, PMID: 33073361

[ref230] Ye M. Wysocki J. William J. Soler M. J. Cokic I. Batlle D. (2006). Glomerular localization and expression of angiotensin-converting enzyme 2 and angiotensin-converting enzyme: implications for albuminuria in diabetes. J. Am. Soc. Nephrol. 17, 3067–3075. doi: 10.1681/ASN.2006050423, PMID: 17021266

[ref231] Yeoh Y. K. Zuo T. Lui G. C. Zhang F. Liu Q. Li A. Y.. (2021). Gut microbiota composition reflects disease severity and dysfunctional immune responses in patients with COVID-19. Gut 70, 698–706. doi: 10.1136/gutjnl-2020-323020, PMID: 33431578 PMC7804842

[ref232] Youngs J. Provine N. M. Lim N. Sharpe H. R. Amini A. Chen Y. L.. (2021). Identification of immune correlates of fatal outcomes in critically ill COVID-19 patients. PLoS Pathog. 17:e1009804. doi: 10.1371/journal.ppat.1009804, PMID: 34529726 PMC8445447

[ref233] Zarkovic N. (2020). Roles and functions of ROS and RNS in cellular physiology and pathology. Cells 9:767. doi: 10.3390/cells9030767, PMID: 32245147 PMC7140712

[ref234] Zeng F. M. Li Y. W. Deng Z. H. He J. Z. Li W. Wang L.. (2022). SARS-CoV-2 spike spurs intestinal inflammation via VEGF production in enterocytes. EMBO Mol. Med. 14:e14844. doi: 10.15252/emmm.202114844 35362189 PMC9081906

[ref235] Zheng Y.-Y. Ma Y.-T. Zhang J.-Y. Xie X. (2020). COVID-19 and the cardiovascular system. Nat. Rev. Cardiol. 17, 259–260. doi: 10.1038/s41569-020-0360-5, PMID: 32139904 PMC7095524

[ref236] Zhou F. Yu T. Du R. Fan G. Liu Y. Liu Z.. (2020). Clinical course and risk factors for mortality of adult inpatients with COVID-19 in Wuhan, China: a retrospective cohort study. Lancet 395, 1054–1062. doi: 10.1016/S0140-6736(20)30566-3, PMID: 32171076 PMC7270627

[ref237] Zuk A. Bonventre J. V. (2016). Acute kidney injury. Annu. Rev. Med. 67, 293–307. doi: 10.1146/annurev-med-050214-013407, PMID: 26768243 PMC4845743

[ref238] Zuo T. Zhang F. Lui G. C. Y. Yeoh Y. K. Li A. Y. L. Zhan H.. (2020). Alterations in gut microbiota of patients with COVID-19 during time of hospitalization. Gastroenterology 159:e8. doi: 10.1053/j.gastro.2020.05.048 PMC7237927 32442562

